# Translation Efficiency and Degradation of ER-Associated mRNAs Modulated by ER-Anchored poly(A)-Specific Ribonuclease (PARN)

**DOI:** 10.3390/cells9010162

**Published:** 2020-01-09

**Authors:** Tian-Li Duan, Han Jiao, Guang-Jun He, Yong-Bin Yan

**Affiliations:** State Key Laboratory of Membrane Biology, School of Life Sciences, Tsinghua University, Beijing 100084, China; duantl12@mails.tsinghua.edu.cn (T.-L.D.); jiaoh17@mails.tsinghua.edu.cn (H.J.); he-gj06@mails.tsinghua.edu.cn (G.-J.H.)

**Keywords:** deadenylation, DNA damage response, ER-anchored ribonuclease, ER-associated mRNAs, mRNA decay, poly(A) length profile, poly(A)-specific ribonuclease, translation efficiency

## Abstract

Translation is spatiotemporally regulated and endoplasmic reticulum (ER)-associated mRNAs are generally in efficient translation. It is unclear whether the ER-associated mRNAs are deadenylated or degraded on the ER surface in situ or in the cytosol. Here, we showed that ER possessed active deadenylases, particularly the poly(A)-specific ribonuclease (PARN), in common cell lines and mouse tissues. Consistently, purified recombinant PARN exhibited a strong ability to insert into the Langmuir monolayer and liposome. ER-anchored PARN was found to be able to reshape the poly(A) length profile of the ER-associated RNAs by suppressing long poly(A) tails without significantly influencing the cytosolic RNAs. The shortening of long poly(A) tails did not affect global translation efficiency, which suggests that the non-specific action of PARN towards long poly(A) tails was beyond the scope of translation regulation on the ER surface. Transcriptome sequencing analysis indicated that the ER-anchored PARN trigged the degradation of a small subset of ER-enriched transcripts. The ER-anchored PARN modulated the translation of its targets by redistributing ribosomes to heavy polysomes, which suggests that PARN might play a role in dynamic ribosome reallocation. During DNA damage response, MK2 phosphorylated PARN-Ser557 to modulate PARN translocation from the ER to cytosol. The ER-anchored PARN modulated DNA damage response and thereby cell viability by promoting the decay of ER-associated *MDM2* transcripts with low ribosome occupancy. These findings revealed that highly regulated communication between mRNA degradation rate and translation efficiency is present on the ER surface in situ and PARN might contribute to this communication by modulating the dynamic ribosome reallocation between transcripts with low and high ribosome occupancies.

## 1. Introduction

Diverse pathways that serve to modulate transcript abundance and translation efficiency dynamically regulate gene expression levels. The rates of transcription, maturation, transportation, and degradation mainly determined the abundance of transcripts [1]. After transported from the nucleus to cytoplasm, the abundance of mature mRNAs is negatively regulated by the decay rate. The bulk of eukaryotic mRNAs are degraded in a deadenylation-dependent pathway, in which deadenylation is the first and rate-limiting step [2,3]. The long poly(A) tails at the mRNA 3′-end are removed by deadenylases, a group of 3′-exonucleases with high specificity of poly(A) as the substrate [1,2,3,4]. Eukaryotic cells generally possess up to a dozen of deadenylases that can be classified into the DEDD and endonuclease-exonuclease-phosphatase (EEP) families [1,4]. Various deadenylases have distinct intracellular functions that are specified by their dissimilar enzymatic properties, binding partners, and subcellular localizations. Most deadenylases have multiple cellular localizations and they can shuttle between the nucleus and cytoplasm. Among them, CCR4 and CAF1 has been observed to exist in processing bodies in some cell types [5,6], poly(A)-specific ribonuclease (PARN) is abundant in the nucleoli and Cajal bodies [7,8], while mammalian PARN-like ribonuclease domain containing 1 (PNLDC1), has been shown to have potential endoplasmic reticulum (ER) distribution [9]. From the developmental view, PNLDC1 is highly expressed during early development to act as a pre-piRNA trimmer [9,10,11], while the other deadenylases are ubiquitous and expressed with high levels in most cell types [1,4]. Abnormality in the expression levels or occurrence of inherited mutations in deadenylases have been linked to human diseases, such as cancer, immune disorder, acute leukemias, developmental delay, bone marrow failure, and dyskeratosis congenital [12,13,14,15,16,17].

Most stable eukaryotic mRNAs have long poly(A) tails of up to ~250 nucleotides [18], which provide multiple binding sites for the poly(A) binding protein (PABP) [19]. PABP affects mRNA stability and translation efficiency in a complicate way, probably depending on its binding partners. During active translation, PABP interacts with the 5′-cap-associated eukaryotic initiation factor and thus contributes to the formation of a closed-loop mRNA to facilitate efficient translation [20,21,22]. At the onset of translation repression, PABP stimulates the deadenylase activity of the PAN2-PAN3 complex or CAF1 and it can enhance miRNA-mediated gene silencing [23,24]. Very recently, PABP was found to bind CCR4 and regulate the deadenylation activity of the CCR4-NOT complex in human cells and yeasts [25,26]. Studies in embryonic cells have suggested that the polyadenylation status of an mRNA is positively coupled to its stability, potency to form a circular structure, assembly of polysomes, and translation efficiency [27,28,29,30,31,32]. This proposal has also been verified in adult mouse heart cells [33]. However, the genome-wide studies challenge this mechanism and suggest that the abundant mRNAs that are highly expressed in somatic cells may have short poly(A) tails to optimize the translational functions [34,35,36]. ER stress has been found to induce an increase in the poly(A) tail length of the ER stress-induced genes, which positively correlates to their de-repression and stabilization. Meanwhile, the repressed mRNAs in the stress-induced RNA granules generally have shorter poly(A) tails [37]. It is possible that the cells utilize both tail-length-dependent and -independent mechanisms to reshape translation globally. It is clear that the change of tail-length contributes to translation regulation despite the controversial opinions in the relationship between poly(A) tail length and translation efficiency.

Translation regulation includes not only the control of gene expression levels, but also the spatiotemporal production of proteins. In eukaryotic cells, the concept of translational compartmentalization has been widely recognized since the groundbreaking work by Palade and his coworkers [38]. In the classical view, ribosomes have two distinct populations: on the ER surface or in the cytosol. The mRNAs encoding membrane, organelle and secreted proteins are sorted to translate on the ER, while the cytosolic and nuclear proteins are produced in the cytosol. The sorting of translation sites is achieved by the recognition of the signaling peptide within nascent proteins by the signal recognition particle (SRP) [39], which delivers on-translation mRNAs to the SRP receptor that is located at the rough ER membrane [40]. Recent findings demonstrate that, besides the canonical SRP co-translational delivering pathway, mRNAs can be conveyed to the ER by an alternative pathway independent of SRP or translation [41,42]. Furthermore, transcriptome and single-molecule studies reveal that a number of mRNAs encoding cytosolic and nuclear proteins associate with the ER in a translation-dependent manner [43,44]. The ER-bound mRNAs have higher ribosome occupancies than those that are retained in the cytosol and they can return back to the cytosol after translation [43].

Although translation is highly localized, the degradation of mRNAs seems likely to occur in the cytosol, since the ER-associated mRNAs are generally occupied by ribosomes and in efficient translation. This leads to an unproved hypothesis that the ER-associated mRNAs need to be released to the cytosol for further deadenylation or degradation purpose. Is it possible for the ER-associated mRNAs to be deadenylated or degraded on the ER surface in situ? Furthermore, can the translation efficiency of the ER-bound mRNAs be re-modulated by the change of tail length but without dissociation from ER? To address these problems, the key is to identify whether there are ER-anchored active deadenylases. Presently, PNLDC1 is the only known deadenylase with potential ER localization [9]. However, PNLDC1 is a pre-piRNA trimmer during early development and it has low abundance in somatic cells [9,10,11]. In this research, we showed that PARN, the homologue of PNLDC1, has obvious ER distribution in the HeLa and HEK-293T cells, as well as in mouse tissues. We found that PARN overexpression reshapes the poly(A) tail length profile of the ER-associated mRNAs, but not the cytosolic mRNAs. Although the ER-anchored PARN has no effect on the global translation efficiency, it can modulate the transcript levels and translation efficiency of a small subset of mRNAs, particularly of genes that are involved in DNA-damage response and cell cycle control. Strikingly, we observed that PARN overexpression significantly reduces the number of transcripts of several mRNAs, but greatly enhances their translation rates by remodeling the polysome profile. These findings shed new lights on the mechanistic understanding of the regulated communications between mRNA decay and translation on the ER surface.

## 2. Materials and Methods

### 2.1. Materials

Rabbit anti-PARN antibody (ab125185 and ab188333) and mouse anti-PDI antibody were purchased from Abcam (Cambridge, MA, USA). Rabbit anti-PARN antibody (A6941) was obtained from Abclonal (Cambridge, MA, USA). Rabbit anti-MAPKAPK-2 antibody (#3042) was from Cell Signaling Technology (Beverly, MA, USA). Mouse antibodies against calnexin, CNOT6, and CNOT7 were purchased from Santa Cruz Biotechnology Inc. (Santa Cruz, CA, USA). Mouse anti-GAPDH antibody was from Bioworld (Louis Park, MN, USA). Rabbit antibodies against LaminA/C, CNOT7, CNOT8, PAN2, MDM2, and RPS3 were from Proteintech (Chicago, IL, USA). The anti-Flag mouse antibody and anti-PNLDC1 rabbit antibody were from Sigma-Aldrich (St Louis, MO, USA). Rabbit antibodies against CDKL1, CCNT2, CLOCK, and MATR3 were purchased from BBI Life Sciences Corp. (Shanghai, China). Horseradish peroxidase (HRP)-conjugated secondary antibodies for Western blot were obtained from Yeasen (Shanghai, China). HRP-labeled sreptavidin for biotin-labeled RNA detection was from Beyotime (Beijing, China). Dylight 488/549/594/649 goat-anti-rabbit/mouse secondary antibodies for immunofluorescence observations were from Bioworld. The transfection reagent Lipofectamine™ and the RNAiMAX Reagent were from Invitrogen (Carlsbad, CA, USA) and Vigofect was from Vigorous (Beijing, China). Doxorubicin hydrochloride (DOX) was from Solarbio (Beijing, China). Cycloheximide (CHX) was from AMRESCO (Solon, OH, USA) and was dissolved in DMSO (Sigma-Aldrich, St Louis, MO, USA). Digitonin, hydroxyurea (HU), urea, and protease inhibitor cocktail used for mammalian cellular extraction were from Sigma. CMPD1 was purchased from Santa Cruz. Recombinant RNasin^®^ ribonuclease inhibitor and sequencing grade modified trypsin were purchased from Promega (Madison, WI, USA). RNase A/T1 Mix and yeast total RNA were from Thermo Scientific (Waltham, MA, USA). NP-40, DNase/RNase-free sucrose and sodium deoxycholate (DOC) were from AMRESCO. Oligo (dT)_25_ cellulose beads for mRNA isolation and the low range ssRNA ladder were purchased from New England Biolabs (Ipswich, MA, USA). Sodium dodecyl sulfate (SDS) and paraformaldehyde were from Merck (Darmstadt, Germany). 1,2-dipalmitoyl-sn-glycero-3-phospho-L-serine (sodium salt) (DPPS) was from Avanti Polar Lipids (Alabaster, AL, USA). The kit for cytosolic and membrane proteins extraction was from KeyGen biotech (Nanjing, China). The kits for RNA 3′end biotin labeling and chemiluminescent nucleic acid detection were from Thermo Scientific. Viewsolid synthesized RNA oligos for PARN knockdown [7]. Takara synthesized Polyadenylate A_20_ and A_200_ was from Sigma. All other chemicals were of analytical grade.

### 2.2. Cell Culture

The HEK-293T and HeLa cell lines were purchased from the China Center of American Type Culture Collection (ATCC, Wuhan, China) and then cultured in the Dulbecco’s modified Eagle’s medium (DMEM, Gibco) with 10% fetal bovine serum (FBS, Gibco, Grand Island, NY, USA) at 37 °C with 5% CO_2_. Plasmids containing the wild-type (WT), the truncated form or site mutated human PARN for cell transfection, were constructed while using pcDNA3.1 (N-Flag), pEGFP-C3, and -N1 vectors. The primers that were used for mutagenesis and plasmid construction were the same as those described previously [16,45,46]. The cells were seeded, transfected using the Lipofectamine™ RNAiMAX Reagent or Vigofect according to the manufacturer’s instructions, cultured for 24 h, and then harvested for further analysis. The knockdown of PARN by siRNA was carried out while using the procedures described previously [7] and the cells were harvested after transfection and then cultured for 72 h. UV treatment was carried out by exposing the cells to UV irradiation for 20 min., and the cells were then cultured in fresh DMEM cell culture medium for 2 h.

### 2.3. Cell Fractionation

Differential detergent fractionation was used to separate the cytosolic and membrane fractions by a fractionation kit that was provided by KeyGen (Nanjing, China). The fractionation was performed according to the manufacturer’s instructions.

Cell fractionation by differential centrifugation after syringe homogenization was carried out while using a 10-cm dish of HEK-293T cells. The cells were washed twice with 10 mL ice-cold phosphate buffered saline (PBS) and then scraped in 1 mL ice-cold PBS with 1 mM DTT and 1× protease inhibitor cocktail. Subsequently, the cells were transferred to a 1.5 mLEppendorf tube and homogenized by a 25-gauge syringe on ice. The homogenate (whole cell lysates) was centrifuged at 1000× *g* for 10 min. to remove unbroken cells, nuclei and cell debris. The supernatant fraction was then centrifuged at 20,000× *g* for 10 min. to remove the large organelles, followed by centrifugation at 100,000× *g* for 60 min. at 4 °C in a Beckman TLA 55 rotor to separate cytosol from microsomes.

Cell fractionation by differential centrifugation after Dounce homogenization was performed while using a 15-cm dish of the HeLa cells. The cells were washed twice with 10 mL ice-cold PBS and then scraped in 4 mL ice-cold homogenate buffer containing 10 mM HEPES-KOH (pH 7.5) buffer, 10 mM KCl, 1 mM MgCl_2_, 1 mM DTT, and 1× protease inhibitor cocktail. The cell suspension was transferred into a pre-cooled 5 mL Dounce homogenizer and homogenized with 15–20 strokes while using the pestle at 4 °C. Subsequently, the homogenates were transferred into a new Eppendorf tube with the addition of 1/10 volume of 2.5 M sucrose to make a 250 mM isotonic solution and then subjected to differential centrifugation. The fractions were obtained by collecting the cell pellets after sequential differential centrifugation of the supernatant fraction, as follows: nucleus, mitochondria, and large membrane fractions were obtained from the pellets after centrifuging at 700× *g*, heavy mitochondria and membrane debris at 3000× *g*, mitochondria, lysosome, peroxisome, and the intact Golgi apparatus at 6000× *g*, mitochondria, lysosome, peroxisome, and the Golgi membrane at 10,000× *g*, lysosome, peroxisome, the Golgi membrane, and large, high-density vesicle from rough endoplasmic reticulum at 20,000× *g*, and all of the vesicles from the endoplasmic reticulum (ER), the plasma membrane, the Golgi membrane, and endosomes at 100,000× *g* for 60 min. All of the fractions were washed with the HM buffer twice and then re-suspended in the RIPA buffer with the addition of 1 × protease inhibitor cocktail.

The isolation of the microsomes and mitochondria was performed while using the published protocols [47]. In brief, a 15-cm dish of the HeLa cells with about 95% consistency was used for the isolation. After homogenization using the pestle to disrupt 80–90% of cells and remove of the nucleus and cell debris by centrifugation at 600× *g* for 10 min. at 4 °C twice, the pellets isolated by centrifugation at 7000× *g* were re-suspended to obtain the Mt0 fraction, further centrifuged at 7000× *g* for 10 min. to obtain the Mt1 fraction, centrifuged at 10,000× *g* to obtain the Mt2 fraction (crude mitochondria) from the pellets. The supernatants and pellets were collected for each step of separation and they were used for further western blot analysis with an equal amount of total proteins.

### 2.4. Extraction of ER-Bound Proteins from Mouse Tissues

ER-bound proteins were extracted from mouse lung, liver, heart, and kidney tissues while using a kit from Bestbio (BB-31454, Shanghai, China). Six to eight-week-old male mice (C57BL/6N) were sacrificed under guidelines and approved by IACUC of Tsinghua University. All of the methods were performed in accordance with the relevant guidelines and regulations. Protease inhibitor cocktail (Sigma) was added to all buffers. 50–100 mg fresh tissues were washed by ice-cold PBS, minced into small pieces, and then washed by ice-cold PBS twice. The tissue cells were lysed with 500 μL buffer A with the addition of PMSF and protease inhibitor cocktail for 10 min. on ice. The cell suspensions were transferred into a clean and pre-cooled 5 mL glass homogenizer and homogenized with 30–40 strokes while using pestle. The tissue homogenates were centrifuged at 1000× *g* at 4 °C. The pellets (nucleus and cell debris) were resuspended in the RIPA buffer, while the supernatants were transferred to a new pre-cooled tube and then centrifuged at 11,000× *g* at 4°C, followed by 50,000× *g* at 4 °C by the TLA-55 rotor (Beckman) for 45 min. to obtain the cytosolic protein enriched fraction from the supernatants. Afterwards, the pellets were washed by 400 μL buffer B, resuspended in 150 μL buffer C on ice for 20 min. to obtain the ER fraction. The BCA kit measured the protein concentration and an equal amount of total proteins was used for Western blot analysis while using rabbit antibody towards PARN (ab188333, Abcam), GAPDH (10494-1-AP, Proteintech), calnexin (10427-2-AP, Proteintech), and laminA/C (10298-1-AP, Proteintech).

### 2.5. Extraction of Membrane Proteins and Trypsin Digestion Assay

The membrane proteins were isolated from the pellet fraction of the 100,000× *g* centrifugation samples that were obtained from the cell fractionation assay described above. The pellets were collected, washed twice with PBS (pH 7.4), re-suspended in PBS, and then divided into five aliquots. The five aliquots were treated with 100 μL PBS buffer without any additions (control), with the addition of 1% Triton X-100, 1% SDS, 0.1 M sodium carbonate (Na_2_CO_3_, pH 11.5), or 1 M sodium chloride (NaCl) for 60 min. After treatment, the samples were centrifuged at 100,000× *g* for 60 min. to separate soluble and insoluble proteins. The pellets were subsequently re-suspended in the same volume of PBS buffer. Trypsin digestion assay were performed to detect membrane protein topology. The pellets of the 100,000× *g* centrifugation samples that were obtained from cell fractionation assay described above were treated by trypsin digestion. The final concentration of trypsin was 25 μg/mL and the mass ratio of protease to total protein was about 1:80. The reaction was conducted at 37 °C for different time intervals ranging from 0 to 60 min. After treatment, the protease inhibitor cocktail was added to each aliquot to terminate the reaction. Subsequently, the samples were centrifuged at 100,000× *g* for 60 min. to separate the soluble and insoluble proteins. After centrifugation, each sample was added with a 5× SDS-PAGE loading buffer and subsequently separated by SDS-PAGE and analyzed by western blot.

### 2.6. Recombinant Protein Expression and Purification

Professor Anders Virtanen kindly provided the recombinant plasmid containing the WT human PARN (p74) (Uppsala University, Uppsala, Sweden). Details regarding mutagenesis, recombinant proteins overexpression in the *Escherichia coli* cells and purification of the recombinant proteins have been described elsewhere [45,46,48]. In brief, the WT and mutated genes were cloned into the vector pET-28a (Novagen, Madison, WI, USA) and verified by sequencing. The recombinant proteins with a His-tag at the N-terminus were overexpressed in *E. coli* BL21 (DE3) (Stratagene, La Jolla, CA, USA) and sequentially purified by Ni^2+^ affinity chromatography while using a 1 mL Ni^2+^ column (GE Healthcare, Madison, WI, USA) and size-exclusion chromatography (SEC) using a Superdex 200 16/60 GL column that was equipped on an ÄKTA purifier (GE Healthcare). The purity of the final products was above 95%, as estimated by SDS-PAGE and SEC analysis. The protein concentration was determined according to the extinction coefficient and *A*_280_ reads. The proteins that were used for biophysical experiments were prepared in 20 mM Tris-HCl buffer, pH 8.0, containing 100 mM KCl, 0.5 mM DTT, 0.2 mM EDTA, and 20% (*v*/*v*) glycerol. Recombinant αB-crystallin and γD-crystallin were purified from the *E. coli* BL21 cell extracts while using the same protocols as those described previously [49,50].

### 2.7. Enzyme Assay

The deadenylase activity was measured by the methylene blue assay or SEC method, as described previously [51,52]. In brief, methylene blue stock solution was prepared by dissolving 1.2 mg methylene blue in 100 mL MOPS buffer (100 mM MOPS-KOH, 2 mM EDTA, pH 7.5), while the stock solution of substrate poly(A) was prepared by dissolving commercial A_200_ or synthesized A_20_ with a concentration of 100 μg/mL. The reaction was initiated by mixing 10 μL enzyme and 40 μL poly(A) stock solution in the standard reaction buffer containing 20 mM Tris-HCl, pH 7.0, 100 mM KCl, 0.5 mM DTT, 0.2 mM EDTA, and 10% (*v*/*v*) glycerol. After 8 min. reaction at 30 °C, methylene blue buffer was added to terminate the reaction and the absorbance at 662 nm was measured while using an Ultraspec 4300 pro UV/Visible spectrophotometer. The SEC assay was performed on a ÄKTA purifier that was equipped with a Superdex 200 10/30 GL column (GE Healthcare). The column was pre-equilibrated for two-column volumes until the UV absorbance and conductance lines were at the same level as that of the control (the standard reaction buffer). The RNA substrate (A_20_ or commercial A_200_) was dissolved in the standard reaction buffer with the addition of 1.5 mM MgCl_2_ and then quantified by measuring the standard curve. The reaction was initiated by mixing 20 μL cell lysate containing 60 μg total proteins and 100 μL substrate stock solutions. After being incubated at 37 °C for a given time, the reaction was quenched on ice. Subsequently, SEC analyzed 100 μL samples and the absorbance at 280 nm, 254 nm, and 215 nm were simultaneously monitored.

### 2.8. Monolayer Surface Pressure Measurements

The classical Langmuir–Blodgett apparatus was used to measure the membrane-binding ability of the purified PARN in vitro [53]. The monolayer surface pressure experiments were performed on an NIMA 9000 (England) Microbalance at 25 °C, as described previously [49]. The monolayer was prepared by spreading phospholipids, phosphatidylethanolamine (PE), phosphatidylserine (PS), or cardiolipin (CL) on the water-air interface of the trough containing 6 mL buffer (20 mM Tris-HCl, pH 8.0, 100 mM KCl, 0.5 mM DTT, and 0.2 mM EDTA). After equilibration, 10–50 μL purified proteins with a final concentration of 20, 50, or 100 nM was injected into the sample loading hole of the trough by the Hamilton syringe. Time-course change in surface pressure was recorded for 6000 s. The critical pressure (π_c_) was calculated by the linear fitting of the changes in surface pressure (∆π) at various initial surface pressures (π_i_) [53]. Besides PARN, the monolayer surface pressure experiments of αB- and γD-crystallins were also performed to take peripheral membrane proteins and cytosolic proteins as the examples of ER.

### 2.9. Liposome Binding Assay

Liposomes were prepared while using the standard method by dissolving DPPS in the chloroform and methanol (3:1) mixture. Subsequently, the organic solvent was removed by rotary evaporation, followed by vacuum pump for about half an hour. The dried lipid film was re-suspended in 20 mM Tris-HCl buffer, pH 7.0, containing 100 mM KCl, 0.5 mM DTT, and 0.2 mM EDTA, and then sonicated to produce small unilaminar vesicles with diameters that ranged from 15 to 50 nm. The quality of the final products was checked by negative-staining electron microscopy. The freshly prepared DPPS liposome was used for the PARN binding assay with a protin:DPPS molar ratio that ranged from 1:50 to 1:200. The final concentration of liposome was 200 μM, while that of PARN ranged from 1 μM to 4 μM. After 1.5 h incubation at room temperature, the mixture was centrifuged at 15,000× *g* for 15 min. at 4 °C. The supernatant and pellet fractions were both analyzed by SDS-PAGE electrophoresis and western blot.

### 2.10. Polysome Profiling

Polysome fractionation was achieved by the standard continuous 10–50% sucrose density gradient centrifugation [54,55]. All solutions, tips, and tubes used for polysome fractionation were RNase-free to avoid RNA degradation. The buffer for the sucrose solutions was 10 mM HEPES-KOH buffer, pH 7.4, containing 5 mM MgCl_2_, 150 mM KCl, 100 μg/mL CHX, 1 mM DTT, RNase inhibitor (40 U/mL), 1× protease inhibitor cocktail, and the detergent NP-40 (*v*/*v* 1%). Layering was conducted while using the polyallomer ultracentrifugation tube (14 × 89 mm, 331372, Beckman).

The whole cell lysate polysome samples were prepared while using a 15-cm dish culturing the HEK-293T cells with 80–90% confluence. The cells were treated with 100 μg/mL CHX for 10 min. at 37 °C, washed with ice-cold PBS containing CHX three times, and lysed in 500 μL cell lysis buffer. The lysates were then transferred to an ice cold, RNase-free Eppendorf tube, incubated for 15 min. on ice, and then centrifuged at 13,000× *g* for 10 min. at 4 °C. The supernatants were collected for further fractionation.

The cytosolic and ER-associated polysome samples were prepared by sequential detergent extraction [56]. After CHX treatment, 1 mL permeabilization buffer (110 mM KCl, 25 mM K-HEPES, pH 7.2, 2.5 mM MgCl_2_, 1 mM EGTA, 0.015% digitonin, 1 mM DTT, 50 μg/mL CHX, 1× Complete Protease Inhibitor Cocktail, and 40 U/mL RNase inhibitor) treated the cells on ice for 5 min., and the soluble fraction (cytosol fraction) was collected. The remaining cells were then washed gently with 5 mL wash buffer (110 mM KCl, 25 mM K-HEPES, pH 7.2, 2.5 mM MgCl_2_, 1 mM EGTA, 0.004% digitonin, 1 mM DTT, and 50 μg/mL CHX) and lysed with 1mL lysis buffer containing 400 mM KCl, 25 mM K-HEPES pH 7.2, 15 mM MgCl_2_, 1% (*v*/*v*) NP-40, 0.1% (*w*/*v*) DOC, 1 mM DTT, 50 μg/mL CHX, 1× Complete Protease Inhibitor Cocktail and 40 U/mL RNase inhibitor on ice for 5 min. The NP-40/DOC soluble fraction (membrane fraction) was collected. The cell debris in the crude cytosolic and membrane fractions were removed by centrifuged at 7500× *g* for 10 min. Nanodrop measured the RNA concentration of the supernatants.

The extracted samples were layered on the top of the prepared sucrose gradients for fractionation by centrifugation at 40,000× *g* for 2 h at 4 °C (Rotor SW41Ti, Beckman, München, Germany). After centrifugation, Fractionator (BIOCOMP) collected the gradient fractions and the UV absorbance at 254 nm was measured simultaneously. Translational efficiency was obtained dividing the sum of the peak area of polysomes with above three ribosomes by that of the monosomes (the 80S subunit). RNAs and proteins in the collected gradient fractions were extracted for further analysis by qRT-PCR, RNA electrophoresis, and Western blot. RNA eletrophoresis was performed by the extraction of RNAs while using the TRIzol reagent, separation using an 8% polyacrylamide gel (in TBE buffer), staining with GelSafe (YPH), and visualization using the ChemiDoc Touch Imaging System (Bio-Rad, Hercules, CA, USA).

### 2.11. Poly(A) Tail Length Determination

The length distribution of the poly(A) tails were analyzed by electrophoresis of the biotinylated poly(A)+ RNAs that were extracted from the total RNAs. In detail, the total RNAs in the cytosolic or membrane fractions were obtained while using the standard methods. The poly(A)+ RNAs were extracted from 500 μg total RNAs using the Oligo(dT)_25_ cellulose beads. After extraction, biotinylation of the 3′-end of poly(A)+ RNAs was performed in a 30 μL reaction solutions containing 8 μL denatured poly(A)+ RNA (~5 μg, 1–50 pmol), 3 μL 10× RNA ligase reaction buffer, 1 μL RNase inhibitor (40U), 1 μL biotinylated cytidine (Bis) phosphate (1 nmol), 2 μL T4 RNA ligase (40 U), and 15 μL 30% PEG at 16 °C overnight. Subsequently, the RNA body of the biotin-labeled RNAs was digested while using the published procedure [57]. In brief, the RNase A/T1 mixture digested the RNA body. The 200 μL reaction solutions contained 12 μL 5 M NaCl, 2.0 μL 0.5 M EDTA, 2 μL RNase A/T1 mixture (2 mg/mL of RNase A and 5000 U/mL of RNase T1), 1 μL total yeast RNA (10 mg/mL) used as the carrier and 153 μL DEPC-treated double distilled water. After 30 min. reaction at 37 °C, the digested RNAs were extracted by TRIzol, which was precipitated by isopropanol and then dissolved in 5 μL DEPC-treated double distilled water. Finally, the length of the RNA body-digested RNAs, which was dominated by the 3′-end poly(A) tail, was determined by 12% polyacrylamide (29:1 acrylamide:bis-acrylamide) separating gel with the addition of 8 M urea and 1× TBE [34,36,57]. The separated RNAs were transferred to a Hybond N+ nylon membrane (GE Healthcare) at 100 V for 1 h, cross-linked twice by ultraviolet at 120 mJ/cm^2^ for 60 s, blocked for 20 min., incubated with the streptavidin-HRP conjugate for 1 h, washed three times, and then used for chemiluminescent detection by the ChemiDoc Touch Imaging System (Bio-Rad, Hercules, CA, USA). An alternative method for determining the poly(A) length distribution was the use of an Agilent RNA 6000 Pico Kit analyzed by the Agilent 2100 bioanalyzer (Palo Alto, CA, USA) through capillary electrophoresis in tiny chips. The Agilent RNA 6000 Pico Kit analysis was performed according to the manufacture’s instructions.

### 2.12. Transcriptome Sequencing

The RNA library was prepared from the total RNAs extracted from the cytosol and membrane fractions of the HEK-293T cell while using the KAPA Stranded RNA-Seq Library Preparation Kit. The samples were sequenced by the Illumina HiSeq X-ten platform at Tsinghua University. The obtained RNA sequences were mapped to the human whole genome (Homo sapiens: HG38) by STAR. The Cuffdiff software analyzed differences in the gene expression profiles. Functional enrichment analysis for the differentially expressed genes was performed while using the PANTHER classification platform of the Gene Ontology Consortium tool (http://geneontology.org/).

### 2.13. qRT-PCR

The total RNAs extracted from the cytosol and membrane fractions of the HEK-293T cell were reverse transcribed to construct the cDNA library while using the standard methods. The qPCR reactions were run with 2× RealStar Power SYBR Mixture (Genstar, Beijing, China). The real-time PCR was performed at 95 °C for 5 min. and followed by 40 cycles of 15 s 95 °C, 20 s at 60 °C, and 35 s at 72 °C. The primers used for the qPCR reactions were as follows: MDM2: forward, 5′-TGCCAAGCTTCTCTGTGAAAG-3′, reverse, 5′-TCCTTTTGATCACTCCCACC-3′; GAPDH: forward, 5′-CGCTCTCTGCTCCTCCTGTT-3′, reverse, 5′-CCATGGTGTCTGAGCGATGT-3′; PDIA3: forward, 5′-TGAGGGATAACTACCGATTTGC-3′, reverse, 5′-TGTATATGCCACAGTCTTGTCC-3′; CDKL1: forward, 5′-AATGTAGAAACAGGGACACGG-3′, reverse, 5′-AGGTTGGGATGCTTGAGTTG-3′; CCNT2: forward, 5′-TGAGATCACCATTGAACACCC-3′, reverse, 5′-CACTGTTGGTTTGTACTGAAGAC-3′; CLOCK: forward, 5′-TCAGTTCAGCAACCATCTCAG-3′, reverse, 5′-GATGTGACTGAGGGAAGGTG-3′; and MATR3: forward, 5′-AGTCTACAAATCCAGCACCAG-3′, reverse, 5′-AGTTTCCACTCTGCCTTTCTG-3′. Quantification was achieved by the determination of the standard curve of *MDM2* by real time PCR and TAE-agarose gel electrophoresis to obtain the copy number per ng of the PCR product.

### 2.14. mRNA Stability

mRNA stability was measured in the HEK-293T cells that were cultured in six-well plates with the DMEM medium containing 10% FBS. Transcription inhibition was accomplished by the addition of 5 μg actinomycin D for 0, 2, 4, 8, or 16 h. After treatment, the cells were fractionated into cytosolic and ER-associated fractions, as described above. The total RNA in the ER-associated fraction was extracted by standard procedures. The amount of *MDM2* mRNA was determined by real time RT-PCR while using PDIA3 as an internal control. The untreated control group normalized the data.

### 2.15. Western Blot

The samples with equal amounts of total proteins ranged from 10 to 60 μg were mixed with 5× loading buffer, boiled, separated by 7.5%, 10%, or 12.5% SDS-PAGE gel, and then transferred to a PVDF membrane (GE). Bound primary antibodies were detected by the HRP-conjugated secondary antibodies (1:3000) while using the SuperSignal West Pico Chemiluminescent Substrate (Thermo Fisher Scientific, Waltham, MA, USA). The PVDF membrane was detected by chemiluminescence and then imaged by ChemiDoc Touch Imaging System (Bio-Rad).

### 2.16. Immunofluorescence

The HeLa cells that were used for immunofluorescence studies were washed twice with PBS (pH 7.4), fixed with 4% paraformaldehyde, penetrated with 0.4% Triton, and then blocked with 10% FBS. The fixed cells were stained with the anti-Flag, anti-PARN, and anti-calnexin antibodies at 4 °C for 14 h. Subsequently, the samples were mounted with Dylight 488 or 594 at room temperature for 1.5 h and then used for the confocal microscopy studies. The nuclei were stained with DAPI. The Imaris software and the Colocalization analysis plugin in Image J were used for colocalization analysis.

### 2.17. Cell Viability

The cells were seeded in a 96-well plate and cultured for 24 h prior to transfection. After 20 h of transfection, the cells were exposed to different stress conditions, including DMSO (control), CMPD1, DOX, and UV irradiation, with various treating time and/or concentrations. After treatment, the wells were moved to fresh DMEM medium with the addition of 10 μL CCK8 reagent each well and then incubated for 1 h at 37 °C. The OD_450_ value was read by a microplate spectrophotometer. The DMEM medium with the addition of the same amount of the CCK8 reagent was used as the blank for spectrophotometer analysis.

### 2.18. Cell Cycle Analysis

The HEK-293T cells were fixed with 70% ethanol in PBS at 4 °C overnight. After washing with PBS three times, the cells were incubated with 100 μg/mL RNase A in PBS for 20 min. at 37 °C and then stained with 50 μg/mL propidium iodide. The percentages of cells in the G_0_/G_1_, S, and G_2_/M phases were determined by FACSCalibur (BD Biosciences, San Jose, CA, USA) and then analyzed with Flowjo 7.6 (Treestar Inc., Ashland, OR, USA).

### 2.19. Ligase Mediated poly(A) tail (LM-PAT) Assay

The ligase mediated poly(A) tail (LM-PAT) assay was performed while using the published protocols [58,59], with some modifications. In brief, the poly(A) tails of 500 ng total RNAs were saturated with 5′-phosphorylated oligo(dT)_16_ at 42 °C in the presence of T4 DNA ligase. An excess amount of oligo(dT) anchor primer (5′-AATGCCAGCTCCGCGGCCGCGTTTTTTTTTTTT-3′) was added to the reaction solutions to anneal at the end of poly(A) tails and then incubated for 2 h at 12 °C to complete ligation. The ligated primers were used to prime RT by M-MLV (Promega). Afterwards, PCR reaction was performed while using the anchor primer as well as a gene specific sense primer. The PCR products in the TBE buffer were resolved on an 8% polyacrylamide gel, stained with GelSafe (YPH), and then visualized by ChemiDoc Touch Imaging System (Bio-Rad). The sequences of the PAT primer of MDM2 is MDM2-primer 1, 5′-GGGTGGATGCTGAATTACATTTTG-3′; MDM2-primer 2, 5′-TTGTGATCATATTGTCTACCATGTAGCCAGCTTTC-3′.

### 2.20. Statistical Analysis

Most of the experiments were performed and analyzed with at least three independent biological replicates, which were separately done while using different sets of cells. The tissue distribution study was performed with two independent experiments and each contained three biological replicates. Statistical analysis of the data was performed by the Student *t*-test or two-way ANOVA test while using GraphPad Prism (GraphPad Software, San Diego, CA) 7.04 or OriginLab 9.0 (OriginLab Corporation, Northampton, MA, USA). A *p*-value less than 0.05 was considered to be significant.

## 3. Results

### 3.1. Substantial Presence of Cytoplasmic PARN on the ER

**Figure 1:**
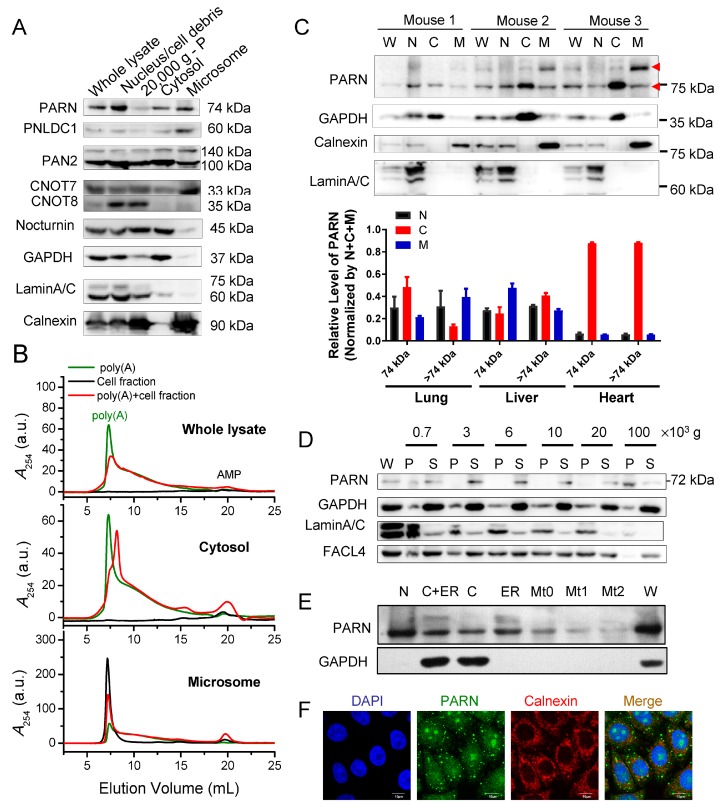
Substantial endoplasmic reticulum (ER)-localization of poly(A)-specific ribonuclease (PARN). (**A**) Subcellular localization of various deadenylases in the HEK-293T cells by Western blot analysis. (**B**) Deadenylase activity of the whole lysate, cytosol and microsome fractions isolated from the HEK-293T cells by the size-exclusion chromatography (SEC) assay. The amount of total proteins in each fraction was kept the same as determined by the bovine serum albumin (BSA) kit. (**C**) Subcellular localization of PARN in mouse tissues fractionated by differential centrifugation. The upper panel shows representative western blot analysis of the lung sample, while the bottom panel shows quantitative analysis of the lung, liver and heart samples (*n* = 3). The red arrows indicate the presence of two forms of PARN identified by the antibody, while the upper one was probably caused by self-association of PARN. (**D**) Western blot analysis of endogenous PARN in various fractions of the HeLa cells obtained by differential centrifugation disrupted by glass homogenizer. Membrane pellets were obtained by 100,000× *g* centrifugation. (**E**) Detection of endogenous PARN in the ER and mitochondria fraction of the HeLa cells. (**F**) Representative confocal images showing the colocalization of endogenous PARN and calnexin in the HeLa cells. PARN was recognized by the monoclonal antibody against PARN (400–500) obtained from Abcam (ab188333). glyceraldehyde-3-phosphate dehydrogenase (GAPDH), LaminA/C, calnexin and FAC4 were used as internal markers of cytosol, nucleus, ER and mitochondria, respectively. W, whole lysate; C, cytosol; M, ER microsomes; N, nucleus; S, supernatant; P, precipitation; Mt0, crude mitochondria fraction; Mt1 and Mt2, mitochondria fraction after one or two steps of purification, respectively. The presented western blot data were representative ones of three independent experiments and the uncropped images are shown in Supplemental Figure S9. See also Supplemental Figures S1 and S2.

The key is to identify active deadenylases on the ER in addressing the problem whether mRNAs could be deadenylated on the ER in situ. We separated the cytosol and ER microsome fractions of HEK-293T cell lysates by differential centrifugation and examined the existence of various deadenylases by commercially available antibodies (Figure 1A). Interestingly, substantial amounts of most deadenylases, except for CNOT8/CAF1b and nocturnin, were found in the microsome fractions. Among them, PARN, PNLDC1, and CNOT7 were enriched in the microsomes. Our results confirmed the previous proposal that PNLDC1 has potential ER localization during the early development of mammalians [9], but the membrane-binding ability of PARN and CNOT7 remains elusive.

The deadenylation activities of the total cell lysates, cytosol, and membrane fractions were evaluated by measuring the degradation of commercial poly(A) substrate and the production of AMP while using the SEC assay and an equal amount of total proteins in each fraction (Figure 1B) [51]. The isolated microsomes exhibited a strong RNA signal that appeared at the void volume of the SEC profile, while a very low absorbance at 254 nm could be detected for the cytosol fraction. This implies that the membrane fraction contained a considerable amount of RNAs that were either enriched in microsomes or well-protected against degradation by various ribonucleases in the cell lysates. After 10 min. incubation, an enhanced AMP signal could be detected for the whole lysates, the cytosolic fraction, and the microsomes. AMP in the degraded samples was more likely generated by the active action of various deadenylases in these cellular extracts while considering that the commercial poly(A) samples did not degrade after incubation under the same conditions. Quite different profiles of the degraded samples were observed for the cytosolic and membrane fractions. A shift of the main peak to a larger elution volume was observed for poly(A) substrate that was degraded by the cytosolic fraction rather than by the microsomes or whole cell lysates. The discrepancy in the size distributions of the degraded poly(A) samples suggested that the cytosolic fraction contained more deadenylases that degraded long poly(A) to moderate lengths with a relatively low efficiency, while the deadenylases in the membrane fraction degraded poly(A) with much higher efficiency without an obvious accumulation of medium length intermediates. Among various deadenylases, the constitutively expressed PARN is unique due to its high catalytic activity against long poly(A) substrate in a processive degradation manner [1,4,60], which coincided with the catalytic behavior of the microsomes. Therefore, we focused this research on the properties and functions of the ER-bound PARN.

We confirmed the ER distribution of PARN in several widely used cell lines and the data that were obtained from the HeLa cells are shown in Supplemental Figure S1A. The transiently overexpressed Flag-PARN showed a similar distribution pattern to endogenous PARN in the HEK-293T cells. The existence of membrane-bound PARN was also detected in mouse tissues, such as lung and liver, but not heart (Figure 1C and Supplemental Figure S1B). It is worth noting that there were two dominant bands for PARN in mouse tissues and sometimes in cell lines, which were confirmed by protein sequence analysis while using mass spectrometry. Previous native-PAGE, cross-linking, DLS, and AFM studies [61,62] have suggested that PARN is capable of forming high-order oligomers both in vitro and in the cells. A close inspection of the confocal microscopic images indicated that the endogenous PARN could form tiny-sized dotted structures in the cytosol (Figure 1F). Therefore, the additional PARN band that appears at a higher molecular weight, which is estimated to be a 148 kDa dimeric form, could be attributed to SDS-resistant PARN forms, which reflects the oligomeric structure of PARN in cells.

The fractionation of the HeLa cell lysates by differential centrifugation indicated that endogenous PARN mainly existed in the nucleus, cytosol and ER fractions (Figure 1D,E), but not in the mitochondria. Exogenously overexpressed PARN have been shown to be mainly located in the nucleus with notable nucleoli accumulation [7]. We compared the confocal immunofluorescence images that were obtained by using three commercially available antibodies against human PARN to avoid misleading observations by the inaccessibility of antibodies to the targeted sequences (Figure 1F and Supplemental Figure S2A). Although the three antibodies showed slightly different amounts of PARN molecules in the nucleus, partial colocalization could be identified between endogenous PARN and the ER marker protein calnexin. Quantification analysis by ImageJ indicated that endogenous PARN positively colocalized with calnexin with a Pearson’s correlation coefficient ranged from 0.107 to 0.338 and a colocalization coefficient of 0.29 ± 0.02 calculated from the data that were stained by the three antibodies (Figure S2B). Thus, the biochemical and immunofluorescence studies both indicated that, besides the predominant distributions in the nucleus and cytosol, PARN also had substantial ER distribution.

### 3.2. C-Terminal Domain Contributes to the Membrane-Binding Ability of PARN

**Figure 2:**
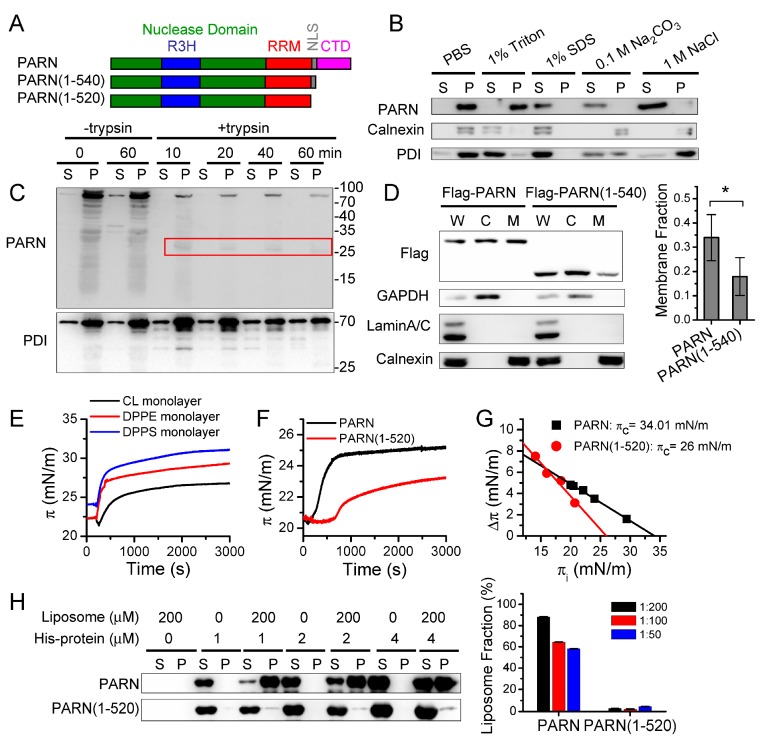
The C-terminal domain is indispensable to the membrane binding ability of PARN. (**A**) Schematic diagram of PARN domain architecture. The two truncated mutants lacking the C-terminal domain (CTD), PARN(1-540) containing the nuclear signal sequence (NLS) and PARN(1-520) lacking NLS were designed for cellular studies and in vitro experiments using purified recombinant proteins. The purified PARN(1-520) had similar structural features to PARN(1-540) as evaluated by spectroscopic methods. PARN(1-520) was used for the in vitro experiments sine PARN(1-540) was prone to degrade in 1 day. (**B**) Membrane-binding properties of PARN. Membrane pellets of the HeLa cells obtained by 100, 000× *g* centrifugation were resuspended in PBS buffer (control), PBS buffer with the addition of 1% Triton, 1% SDS, 0.1 M Na2CO3 (pH 11.5), or 1 M NaCl and equilibrated at 4 °C for 60 min. The supernatant (S) and precipitation (P) fractions were separated by ultra-speed centrifugation and used for Western blot analysis. Calnexin and PDI were used as the references for ER integral membrane proteins and ER lumen proteins, respectively. (**C**) Trypsin digestion assay. Membrane pellets of the HeLa cells were resuspended in PBS buffer and incubated in the presence of 25 μg/mL trypsin at 37 °C. PDI was used as a reference. The red box indicates the position of a trypsin-resistant fragment corresponding to the CTD. (**D**) Western blot analysis of the effect of CTD deletion on the ER-localization of PARN in the HeLa cells. W, whole cell lysates; C, cytosol; M, ER microsomes. Quantitative data (*n* = 3) are shown in the right panel. The membrane fraction was calculated by dividing the protein amount in the membrane fraction by the sum of proteins in the cytosolic and membrane fractions. * *p* < 0.05 (**E**) Membrane insertion ability of PARN into monolayer membranes spread by cardiolipin (CL), 1,2-dipalmitoyl-sn-glycero-3-phosphoethanolamine (DPPE), and 1,2-dipalmitoyl-sn-glycero-3-phospho-L-serine (DPPS). (**F**) Removal of the CTD impairs PARN insertion into the DPPS monolayer. (**G**) Critical surface pressure (*π*_c_) determined by the intersection at the X-axis for the linear relationship between initial surface pressure (*π*_i_) and changes in surface pressure at each *π*_i_. (**H**) Liposome binding assay. The full length and truncated PARN were mixed with liposome generated using DPPS and incubated at room temperature for 1.5 h. The liposome-bound PARN was detected by western blot. The right panel shows the quantitative analysis results (*n* = 3) and bars with different colors represent the labeled molar ratios between PARN and liposome. The presented western blot data were representative ones of three independent experiments and the uncropped images are shown in Supplemental Figure S9. W, whole lysate; C, cytosol; M, ER microsomes; S, supernatant; P, precipitationl.

The binding property of PARN with the ER membrane was investigated by the tolerance of membrane-bound PARN to extreme conditions, including detergents, high concentrations of salt, and high pH (Figure 2B). PARN could be disassociated from the membrane by treating with 1% SDS, 0.1 M Na_2_CO_3_ (pH 11.5), or 1 M NaCl, but not by PBS or 1% triton. PARN was not an integral membrane protein or luminal protein according to its different behaviors from calnexin or PDI. PARN was more likely to be a peripheral membrane protein bound with ER membrane via ionic interactions since 0.1 M Na_2_CO_3_ (pH 11.5) or 1 M NaCl can release peripheral proteins.

Trypsin digestion assay was used to identify the orientation and potential domain/region that is responsible for the membrane binding of PARN (Figure 2C). The microsome-bound PARN could be quickly digested by trypsin in a time-dependent manner, while the luminal protein PDI was degraded much slower. A cleavage fragment with a molecular weight of ~25 kDa was quite resistant to trypsin treatment and it could be identified by antibody recognizing PARN(589–639). This region was in the C-terminal domain (CTD) of PARN, which has been shown to be predominantly intrinsically disordered [8]. The resistance of this disordered fragment against trypsin digestion suggested that the CTD might be protected via membrane binding. This proposal was further verified by the observation that the removal of the CTD significantly reduced the membrane distribution of PARN in the HEK-293T cells (Figure 2D).

PARN can associate with the ER membrane via interacting with the lipids or integral membrane proteins. Monolayer surface pressure (π) measurements and liposome binding analysis of purified recombinant proteins were used to elucidate this problem. The purified PARN exhibited a strong potency to insert into the Langmuir monolayer spread while using DPPS, DPPE, or CL, as evaluated by the abrupt increase of surface pressure after the injection of the protein solutions and large change in surface pressure when the system reached equilibrium (Figure 2E). Quantification analysis was performed while using monolayer spread by DPPS, one of the main negatively charged phospholipids in the ER since PARN showed little lipid-type-dependence [63]. The critical surface pressure (π_c_) of the full-length PARN was 34 mN/m, which was within the physiological surface pressure ranging from 31 to 34 mN/m. αA- and αB-crystallins, which have been shown to be ER peripheral membrane proteins [64], had π_c_ values of 31 mN/m [49] and 23 mN/m (Supplemental Figure S3), respectively. As the control, the highly soluble cytosolic protein γD-crystallin had a π_c_ value of approximately 15 mN/m (Supplemental Figure S3).

The removal of CTD dramatically impaired the membrane-insertion ability of PARN by decreasing π_c_ to ~26 mN/m. Furthermore, the full-length PARN, but not the truncated mutant PARN(1-520), could be deposited together with the DPPS liposome. Almost all of the PARN molecules were in the liposome fraction, when the PARN:DPPS molar ratio reached 1:200 (Figure 2H). Although the recruitment by ER integral membrane proteins could not be excluded, the in vitro assays using purified proteins suggested that PARN had the intrinsic ability to bind with the ER membrane through the intrinsically disordered CTD.

### 3.3. Ser557 Phosphorylation Modulates the ER Localization of PARN to Respond to DNA Damage Response (DDR)

**Figure 3:**
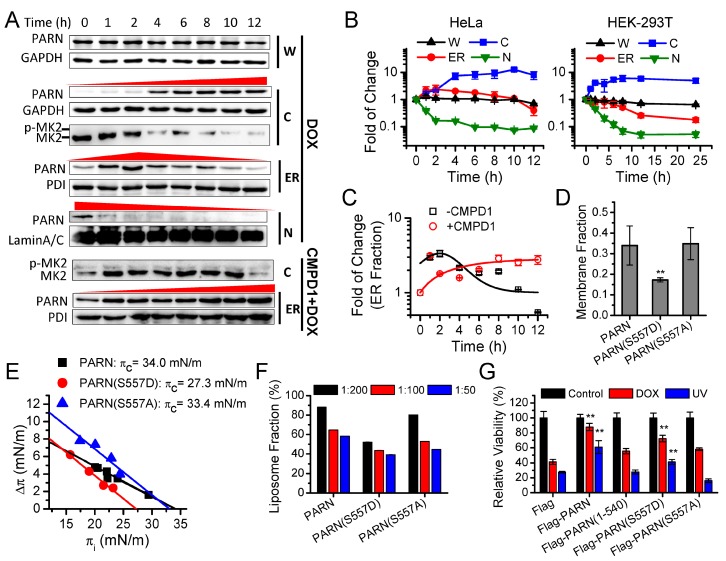
Ser557 phosphorylation contributes to the subcellular translocation of PARN induced by genotoxic stresses. (**A**) Time-course study of PARN subcellular distribution after treating the HeLa cells by 10 μΜ doxorubicin to induce DNA damage. GAPDH, PDI, and lamin A/C were used as the marker proteins for cytosol, ER and nucleus. The bottom panel shows the changes of ER-anchored PARN and cytosolic MK2 for cells pre-treated with MK2 specific inhibitor CMPD1. The presented data were representative ones of three independent experiments and the uncropped images are shown in Supplemental Figure S9. (**B**) A comparison of PARN translocation between the HeLa cells and HEK-293T cells. The protein level were normalized by the value measured at time=0 h for each fraction (*n* = 3). (**C**) Quantitative analysis of the effect of CMPD1 pre-treatment on the change of ER-anchored PARN under genotoxic stress in the HeLa cells (*n* = 3). The raw data in the absence of CMPD1 were fitted by a peak function, while those in the presence of CMPD1 were fitted by a single exponential function. (**D**) Effect of S557D and S557A mutations on ER-anchored PARN in the HEK-293T cells. S557D was used to mimic Ser557 phosphorylation by MK2, while S557A was used as a negative control to mimic the dephosphorylated form (*n* = 3). Representative western blot analysis is shown in Supplemental Figure S4C. (**E**) S557D but not S557A reduced the ability of purified PARN to insert into DPPS monolayer evaluated by surface pressure experiments. (**F**) S557D reduced the ability of purified PARN to bind with DPPS liposome (*n* = 3). Bars with different colors represent the labeled molar ratios between PARN and liposome. Representative western blot analysis is shown in Supplemental Figure S4D. (**G**) Effects of exogenously overexpressed PARN on cell viability for cells that were treated by doxorubicin or UV (*n* = 3). The presented data are shown as mean ± SD. ** *p* < 0.01 when compared with the corresponding data in the Flag group. See also Supplemental Figure S5. W, whole lysate; C, cytosol; ER, ER microsomes; N, nucleus.

PARN is involved in diverse physiological and pathological processes [60,65]. We performed a screen of various stress conditions, including serum deprivation, oxidative stresses, and genotoxic stresses in the HEK-293T cells, to elucidate the potential cellular functions of the ER-anchored PARN (Supplemental Figure S4A). We found that only genotoxic conditions could induce obvious changes in the ER distribution of PARN. We then tracked the time-course change in the protein level and cellular distribution of PARN after treating the HeLa (Figure 3A) and HEK-293T cells (Supplemental Figure S4B) by doxorubicin (DOX) to induce DNA damage. No significant changes in the protein levels of PARN were found in both of the cell lines, implying that *parn* transcription was not responsive to DNA damage. Dramatic translocation of PARN from the nucleus to cytoplasm was observed in both cell lines (Figure 3B) when treated by DOX, but not the DNA damage-mimic reagent hydroxyl urea [8]. In the HeLa cells, the amount of ER-anchored PARN abruptly increased within 2 h DOX treatment, followed by a slow decline. The distinct time-dependence of the ER-anchored PARN after DOX treating suggested that the change in the amount of ER-anchored PARN was not a side-effect of nuclear-cytoplasmic translocation, but it was more likely to result from active regulation of the cells during DDR. In the HEK-293T cells, the lack of an apex value suggested that the ER localization of PARN was differently regulated in the two types of cell lines.

While considering that purified PARN itself had strong membrane-binding ability in vitro, a possible way to modify its ER association was post-translational modifications. Previously, it has been reported that Ser557 of PARN can be phosphorylated by MAPKAP kinase 2 (MK2) in the U2OS and HeLa cells under genotoxic stresses [66]. A comparison of MK2 status indicated that MK2 was activated to the phosphorylated form shortly after DOX treatment and then declined in the HeLa cells, whereas MK2 in the HEK-293T cells maintained in the inactive dephosphorylated state (Figure 3A).

The MK2 status and PARN distribution were determined in the HeLa cells pre-treated with CMPD1, a p38α inhibitor that specifically blocks the activation of MK2, to verify the proposal that PARN could be dissociated from the ER by MK2-induced phosphorylation [67,68]. CMPD1 completely blocked MK2 phosphorylation in the HeLa cells (Figure 3A) and meanwhile the ER-anchored PARN continuously elevated after DOX treatment (Figure 3C). This suggested that MK2 was a negative regulator of the ER-localization of PARN. Furthermore, the substitution of Ser at position 557 by Asp, but not Ala, significantly reduced the ability of PARN to bind with the ER membrane in the HEK-293T cells (Figure 3D), to insert into the DPPS monolayer (Figure 3E) or bind with the DPPS liposome (Figure 3F).

The above experiments clearly indicated that Ser557 phosphorylation by MK2 negatively regulates the ER distribution of PARN, which could explain the slow decline of ER-bound PARN in the HeLa cells (Figure 3A–C). However, PARN also exhibited dissociation from the ER after DNA damage, although the MK2 status was unchanged in the HEK-293T cells (Supplemental Figure S4). Therefore, MK2 is more likely to be one of the regulators of PARN translocation. Furthermore, it remains elusive for the mechanism that underlies the increase of ER-localized PARN immediately after DOX treatment. This increase was not due to the translocation of the nuclear PARN to the cytosol observed in both of the HeLa and HEK-293T cells, since only an apex was only observed in the HeLa cells. Further research is needed for identifying additional regulators that modulate the ER-binding of PARN.

PARN overexpression significantly protected the HEK-293T cells against genotoxic stresses and facilitated cell survival (Figure 3G). The S557D mutation reduced the protective effect of PARN, while the removal of the CTD or mutation by S557A completely abolished the viability enhancement that was introduced by overexpressed PARN. The ER-cytosolic shuttle that was modulated by Ser557 phosphorylation seems to be important to the functions of PARN in cell survival under genotoxic conditions. Although the cytoprotective effect of PARN might involve functions in the nucleus and cytoplasm, the disturbance of shuttle between the ER and cytosol might impair the step-wise function of PARN in the cytoplasm during DDR.

### 3.4. ER-Anchored PARN Modulates poly(A) Tail Length Distribution of ER-Associated mRNAs but Does Not Affect Global Translation Efficiency

**Figure 4:**
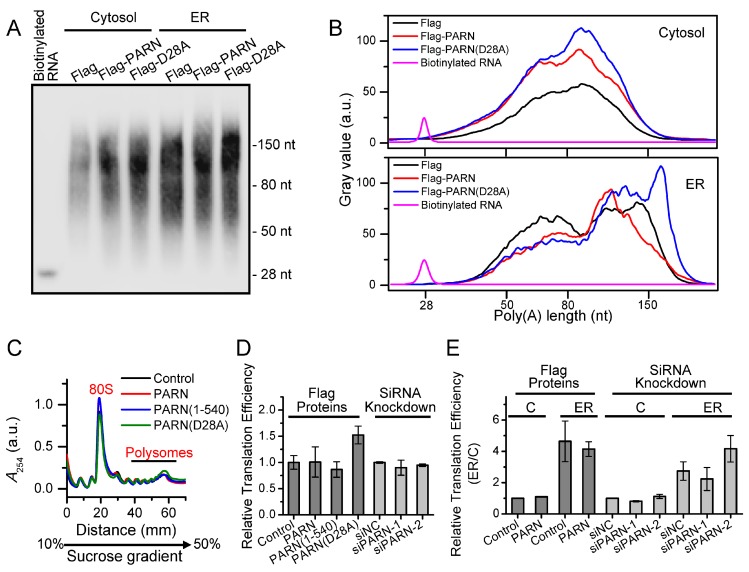
Overexpression of PARN reshapes the poly(A) tail length distribution of ER-associated mRNAs but has no impact on global translation efficiency in the HEK-293T cells. (**A**) Representative Northern blot analysis of the poly(A) tail length distribution of poly(A)+ RNAs with 3′-end biotin labeling and RNA body digestion (*n* = 3). One repetition run on the same gel is shown in Supplemental Figure S9. ER-associated RNAs were extracted from the ER fractions of HEK-293T cells with overexpressed Flag-PARN using oligo(dT)_25_, and therefore short poly(A) with length shorter than 25 nt might be partially lost during extraction. Cells that were transfected with plasmids containing Flag were used as the control. (**B**) Poly(A) tail distribution profiles obtained by measuring the gray values of the plot shown in panel A. The other biological replicates exhibited similar patterns to the presented one. (**C**) Representative polysome profiling analysis of the ER fraction extracted from the HEK-293T cells with overexpressed PARN, PARN(1-540), or PARN(D28A). (**D**) The effect of PARN overexpression and knockdown on global translation efficiency of the whole cell lysates. The translation efficiency was evaluated by the ratio between polysomes and monosomes. The presented data were normalized by the controls (*n* = 3–6). Two siRNA sequences were used to knockdown PARN and denoted as siPARN-1 and siPARN-2, respectively. (**E**) Effect of PARN overexpression and knockdown on translation efficiency of the cytosol and ER fractions. The presented data were normalized by the value of the cytosol fraction of the control group (*n* = 3). C, cytosol; ER, ER microsomes.

The deadenylase activity of the ER-anchored PARN was evaluated by determining the global changes in poly(A) length distribution of ER-associated and cytosolic mRNAs that are induced by overexpressed Flag-PARN or Flag-PARN(D28A) in the HEK-293T cells. Under our experimental conditions, the poly(A) tails of the ER-bound mRNAs had two major populations that were centered at around 140 nt and 65 nt, while the cytosolic RNAs were dominated by moderate lengths that were centered at around 90 nt (Figure 4A,B). It remains unclear for the discrepancy in poly(A) tail length distribution between the cytosolic and ER-associated mRNAs. A possible explanation is that certain poly(A) lengths may be required for the localization and efficient translation on the ER. PARN overexpression did not significantly affect the poly(A) tail length distribution of the cytosolic mRNAs, which implied that PARN might not be the dominant deadenylase in the cytosol. As for the ER-associated mRNAs, PARN narrowed down the poly(A) tail length distribution by shifting the centers of the two populations to around 120 nt and 70 nt. In contrast, the dominant negative mutant PARN(D28A) was competed with endogenous PARN and greatly increased the abundance of long poly(A) tails with lengths above 150 nt. A comparison of the opposing actions between the WT PARN and the PARN(D28A) suggested that the endogenous ER-anchored PARN might prefer to degrade long poly(A) tails, but it had little impact on the medium length and short poly(A) tails. The underlying mechanism of this observation remains elusive and it needs further investigation. The capillary electrophoresis analysis using the Agilent RNA 6000 Pico Kit confirmed the above results, although the resolution was much lower than the traditional gel analysis (Supplemental Figure S5).

Polysome profiling was performed to detect whether changes in the poly(A) tail length distribution affected global translation efficiency (Figure 4C). No significant changes in the polysome to monosome ratio (P/M) were observed for the HEK-293T cells overexpressing the full length PARN, PARN(1-540), or PARN(D28A) and cells with the knockdown of endogenous PARN by siRNA (Figure 4D). We further profiled the translation of the ER and cytosolic transcripts separately (Figure 4E). The ER fractions had a P/M value 2–5-fold larger than the cytosolic fraction, which is consistent with previous observations that the ER-associated mRNAs preferred to binding with more ribosomes than the cytosolic mRNAs [43,69]. Neither overexpression nor knockdown of PARN had any significant impacts on the global translational efficiency for both of the ER and cytosolic fractions. A combined analysis of the change in poly(A) tail length distribution (Figure 4B) and translation efficiency (Figure 4E) suggested that ER-bound PARN might have a non-specific action of shortening long poly(A) tails beyond the scope of efficient translation. Our results also reinforced the emerging concept that the poly(A) length distribution is not always correlated with the global translation status in somatic cells [34,35,36].

### 3.5. ER-Anchored PARN Modulates the Decay of a Small Subset of ER-Bound mRNAs

**Figure 5:**
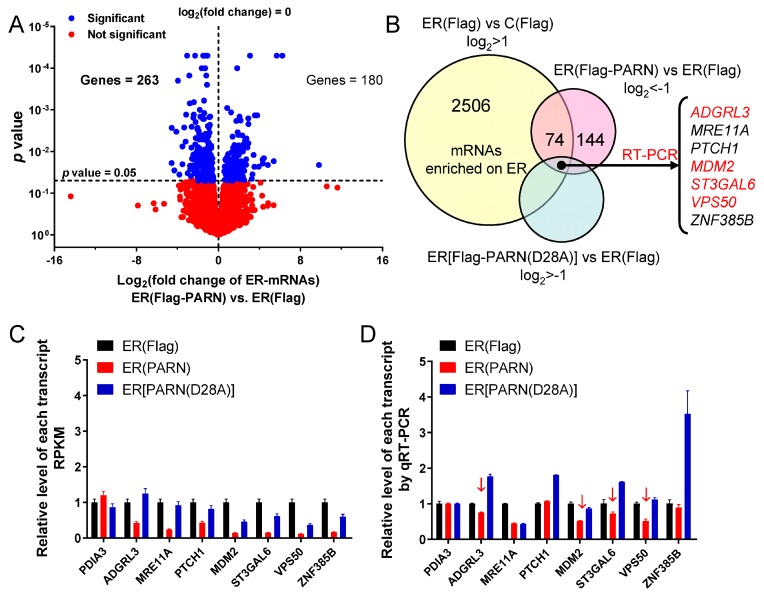
Targets of ER-anchored PARN screened by transcriptome sequencing. (**A**) The volcano plot of fold change and *p* value for transcripts in the ER fraction of the HEK-293T cells overexpressing PARN vs. the control cells transfected with the empty vector. (**B**) The selection strategy to figure out the ER-enriched targets of PARN. Four out of seven were further verified by qRT-PCR and labeled in red. (**C**) The Reads Per Kilobase per Million (RPKM) values of the candidate transcripts in the ER fractions obtained from transcriptome sequencing. (**D**) Relative abundance of the candidate transcripts in the ER fraction determined by qRT-PCR. See also Supplemental Figure S6.

Transcriptome sequencing was used to screen out the targeted genes of ER-anchored PARN by comparing the transcripts that were enriched in the ER fraction between cells transiently transfected by control plasmids containing Flag and plasmids containing Flag-PARN (Figure 5A). Sequencing analysis yielded 13,200 transcripts that could mapped to human genome map, among which 2506 and ~3000 transcripts were predominantly enriched in the ER and cytosolic fractions, respectively. The abundance of the remaining 7700 transcripts had no significant differences between the ER and cytosolic fractions, which implied that the bulk of transcripts shuttled between the ER and cytosol. Only transcripts enriched in the ER fraction were used for further filtering to avoid misleading results that are induced by the action of cytosolic PARN. Meanwhile, we chose the enzymatically inactive mutant D28A as the negative control to avoid the introduction of multiple variables by the modification of protein-protein interaction network caused by the S557D mutant. Prior to sequencing analysis, the expression of the WT and mutated PARN were adjusted to the same level by western blot analysis.

74 transcripts highly enriched in the ER fraction were screened out to have positive response to overexpressed PARN while using the selection strategy shown in Figure 5B. The candidate targets of ER-anchored PARN were further narrowed down by filtering off the transcripts downregulated in the ER fraction of cells overexpressing the inactive mutant PARN(D28A). We obtained seven mRNAs by this approach (Figure 5C and Supplemental Figure S6). Note that PARN might have much wider spectrum of targets that translated on the ER, but they were filtered off due to the lack of significant enrichment in the ER fraction or interference of endogenous PARN. Four among the seven targeted mRNAs were finally identified by experimental validations while using qRT-PCR (Figure 5D) and mRNA decay kinetics (Supplemental Figure S6C). Three among the four mRNAs encodes membrane proteins: adhesion G protein-coupled receptor L3 (ADGRL3) is an integral membrane protein that is localized on the plasma membrane of neuron cells, type 2 lactosamine alpha-2,3-sialyltransferase (ST3GAL6) is a transmembrane protein localized on the Golgi apparatus and VPS50 is an endosome protein that is involved in endocytic recycling. Further research is needed to elucidate whether PARN is involved in the regulation of endomembrane system. *MDM2* encodes a well-known E3 ubiquitin-protein ligase that acts as a negative regulator of p53/TP53 stability by mediating its ubiquitination and degradation in proteasome. According to the functions of these genes, we focused our study on *MDM2*, since MDM2 has been well validated in the p53-dependent DDR pathway, coincident with the potential cellular functions of the ER-anchored PARN that is shown in Figure 3.

### 3.6. ER-Anchored PARN Modulates DNA Damage Response by Affecting the Translation Efficiency of MDM2

**Figure 6:**
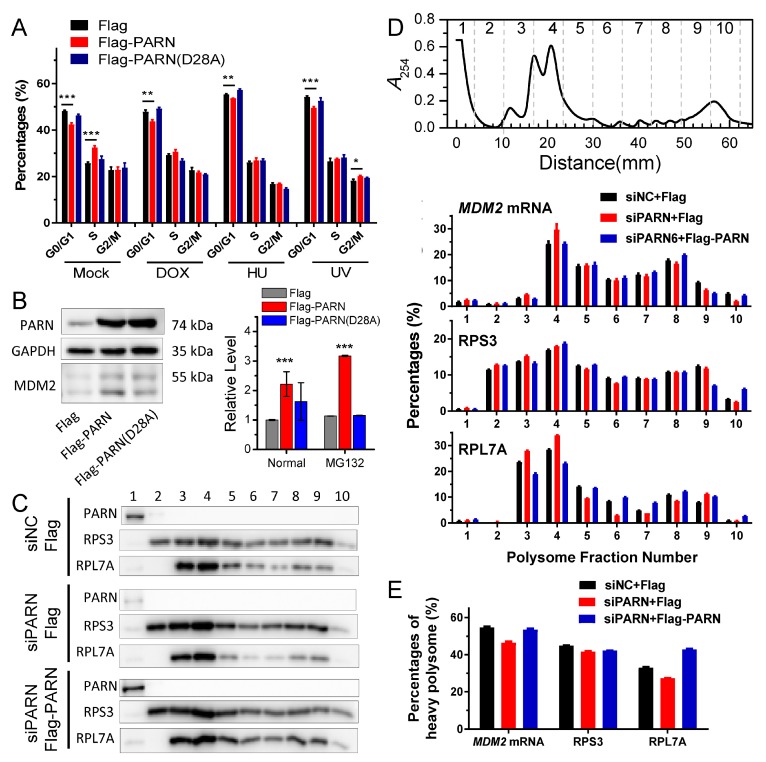
ER-anchored PARN enhances MDM2 translation efficiency by increasing the proportion of heavy polysomes in the HEK-293T cells. (**A**) Cell cycle analysis of the HEK-293T cells overexpressing PARN and PARN(D28A) by flow cytometer under normal and various genotoxic conditions. The flow cytometer data were analyzed by Flowjo 7.6 to calculate the percentage of cells in the G0/G1, S, and G2/M phases (*n* = 3). (**B**) The effect of PARN overexpression on MDM2 protein level. GAPDH and actin were used as the loading control for the normal cells and cells treated by MG132, a proteasome inhibitor. The left panel shows the representative Western blot analysis data for cells cultivated under normal conditions. The right panel shows the results of quantitative analysis by Image J for MDM2 level in cells that were cultivated under normal conditions (*n* = 7) and treated by 10 μM MG132 for 2 h (*n* = 3). (**C**) Western blot analysis of the protein levels of PARN, RPS3, and RPL7A in each fraction of the polysome profiles of cells with the knockdown of PARN by siRNA and PARN-knocked down cells with the rescue of PARN. (**D**) Quantitative analysis of the *MDM2* mRNA level, the RPS3 and RPL7A protein levels in each fraction of the ER polysome profiles (*n* = 3). The copy number determined the *MDM2* mRNA levels. (**E**) Percentages of the *MDM2* mRNA, RPS3 and RPL7A in heavy polysomes (*n* = 3). * *p* < 0.05, ** *p* < 0.01, *** *p* < 0.001. The presented western blot data were representative ones of at least three independent experiments and the uncropped images are shown in Supplemental Figure S9.

The down-regulation of *MDM2* mRNA level is expected to release more p53 molecules to induce cell cycle arrest in the cancer cells [70]. Surprisingly, the overexpression of the wild type PARN, but not the inactive mutant significantly reduced the percentages of the HEK-293T cells in the G_0_/G_1_ phase under both normal and genotoxic conditions (Figure 6A and Supplemental Figure S7A). The protein level of MDM2 was enhanced about two-fold in the HEK-293T cells overexpressing PARN when cultivated under normal conditions and about three-fold in the cells with the inhibition of proteasome activity by MG132, in contrast to the reduction in steady-state mRNA level and acceleration in *MDM2* mRNA decay (Figure 6B). The opposing effects of overexpressed PARN on mRNA and protein levels were further examined while using several potential targets of PARN in the cytosol (Supplemental Figure S7B). The results that are shown in Figure 5 and Figure S6 indicated that the bulk of mRNAs shuttled between the ER and cytosol, and most of the previously identified PARN had no preference of ER or cytosol location. The potential cytosolic targets of PARN were chosen with a predominant cytosol location, a high RPKM score, and the availability of antibody to avoid misleading by the action of ER-anchored PARN. Based on these criteria, we identified four mRNAs as potential PARN cytosolic targets, which have not been previously reported, probably due to the dissimilar gene expression profiles of different cell lines. Three among the four proteins showed little alterations in their protein levels, while CDKL1 was upregulated. Intriguingly, CDKL1, which is a member of the cyclin-dependent kinase-like (CDKL) family, has been reported to play a role in carcinogenesis [71]. These observations were consistent with the above results that PARN had little impact on the global translation efficiency of the cells (Figure 4). It seems that the action of PARN was highly regulated and it only triggered the decay of a small subset of transcripts, particularly those involved in DDR and cell cycle control.

The opposing effects on MDM2 mRNA and protein levels led to the proposal that PARN might modulate *MDM2* translation efficiency on the ER. PARN did not affect the poly(A) tail length distribution of *MDM2* (Supplemental Figure S7C), which implied that the modification of *MDM2* translation efficiency by PARN did not depend on the length of the poly(A) tail of the transcript. PARN did not associate with the translation machineries on the ER, since it was absent from the assembled ribosomes or polysomes (Figure 6C and Supplemental Figure S8A). Polysome profiling combined with qRT-PCR was applied to evaluate the amount of transcripts in each fraction of the polysome profiles (Figure 6D and Supplemental Figure S8B). PARN overexpression induced a shift of the distribution of *MDM2* transcripts to heavy polysomes. Meanwhile, ribosomes were also translocated towards heavy polysomes, as evaluated by the levels of RPS3 and RPL7A (Supplemental Figure S8C), which are components of the 40S and 60S ribosomal subunits, respectively. In contrast, the knockdown of endogenous PARN by siRNA had an opposing effect by decreasing the proportion of *MDM2* and RPL7A in polysomes. Rescue experiments indicated that the overexpression of Flag-PARN in the PARN-deficient cells could re-distribute *MDM2* transcripts, RPS3, and RPL7A in the polysomes (Figure 6E). Therefore, PARN probably promotes the degradation of *MDM2* transcripts with low ribosome occupancies, facilitates the reallocation of the released ribosomes from the degraded transcripts to heavy polysomes, and thereby might result in an enhancement in translation efficiency, even though the steady state mRNA level of *MDM2* is downregulated. The ribosome reallocation could also explain why no significant changes in the protein levels were observed for some of the PARN targets (Supplemental Figure S7B).

## 4. Discussion

The concept that translation is spatiotemporally regulated has been well-documented since the early 1970s [38]. The compartmentalization of mRNAs and ribosomes involves two predominant localizations, the ER surface, and cytosol. ER-associated mRNAs are believed to translate with a high efficiency and translation repression will release the ER-bound mRNAs back to the cytosol [43,44,69]. It is worth noting that, during ER stress, IRE1, an ER-localized endoribonuclease, can be activated to splice *XBP1* mRNA to a translatable form [72], which implies that the ER surface might provide an interface not only for efficient translation, but also for prerequisite translation regulation. Herein, we further asked whether ribonucleases could target the ER-bound mRNAs and modulate their decay and translation efficiency in situ on the ER surface.

It is difficult to track the fates of ER-bound mRNAs since the bulk of mRNAs shuttled between the ER surface and cytosol (Figure 5). We then addressed this problem by identifying ER-anchored deadenylases exhibiting deadenylation activities in somatic cells and by verifying the membrane-binding ability of PARN in vitro (Figure 1 and Figure 2). We showed that the ER-anchored PARN requisitely orchestrated translation by modulating the poly(A) length distribution and mRNA abundance on the ER. Our findings shed new lights on the mechanistic understanding regarding the emerging concept of crosstalk between mRNA degradation and translation by showing that this crosstalk can occur not only in cytosol, but also on the ER surface, the workplace of highly efficient translation.

Besides PARN, our results also indicated that the other types of deadenylases might also have ER distributions. Further research is needed to verify this hypothesis. An unresolved question is the fate of the deadenylated mRNAs that are generated by the action of ER-anchored deadenylases. Are these deadenylated ER-associated mRNAs released to the cytosol or degraded by the decay machinery on ER in situ? Furthermore, will the poly(A) length affect the ER-localization of mRNAs? Further study using single-molecule RNA imaging in living cells might provide clues to these unresolved problems.

Among various deadenylases, PARN is evolved later than the other deadenylases and it only exists in vertebrates and higher plants [1,4,60]. The transcriptome-level study has revealed that basal deadenylation in human cells is unaffected by the depletion of PARN [25]. Our results showed that PARN overexpression did not affect the poly(A) length profile of cytosolic mRNAs, while the length distribution of the ER-bound mRNAs was reshaped (Figure 4). Transcriptome analysis indicated that only a small portion of the ER-bound mRNAs had significant changes in their abundance (Figure 5). A combined analysis of the results that are shown in Figure 4 and Figure 5 suggested that the ER-associated PARN triggered the decay of only a small subset of ER-bound transcripts, while it might play a non-specific global role in the shortening of long poly(A) tails without the destabilization of most ER-bound mRNAs. PARN had little impact on the poly(A) length distribution of cytosolic mRNAs, which implied that the basal deadenylation in the cytosol is probably achieved by the PAN2-PAN3 and CCR4-NOT complexes but not PARN. Consistently, it has been reported that PARN knockdown in mouse myoblasts affects the stability of a discrete subset of cell-mobility related mRNAs [73].

Intriguingly, PARN can modulate the decay of mRNAs encoding proteins that are crucial to cell cycle regulation in both the ER and cytosol fractions. The importance of PARN in cell cycle progression has been revealed by its essential role during early development in both vertebrates and higher plants [1,60] and the proliferation of several types of cancer cells [12,16,17]. PARN can modulate cell cycle progression by targeting a couple of RNAs, including *Gadd45α* [66], *p21* [16], *p53* [16,74], genes that are involved in telomere maintenance [75], miR-21 [76], miR-125b [77], and multiple miRNAs repressing p53 [78]. Although it is clear that PARN activity is highly regulated to precisely control cell cycle progression, it remains elusive as to how PARN defines its targets.

**Figure 7:**
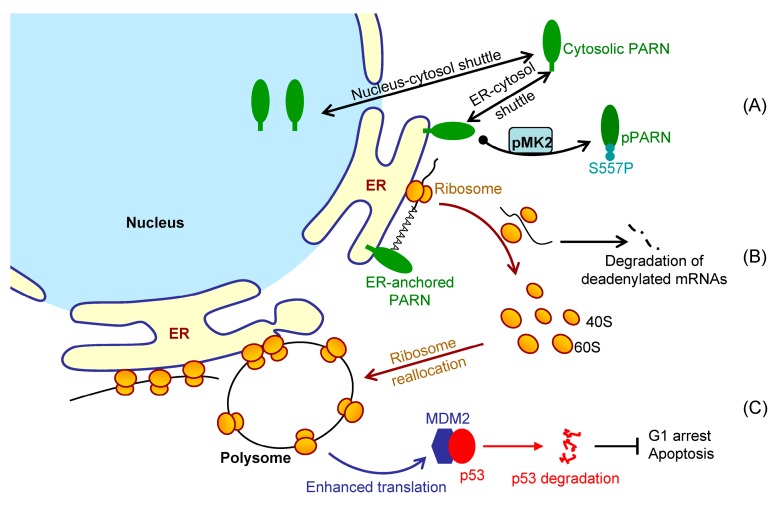
A working model for the potential functions and regulation of ER-anchored PARN. (**A**) PARN has multiple cellular localizations and translocation is mediated by posttranslational modifications. The C-terminal domain contributes to the ER association of PARN. Ser557 phosphorylation by MK2 dissociates PARN from the ER during the late stage of DNA damage response. (**B**) ER-anchored PARN reshapes the poly(A) tail length distribution of ER-associated mRNAs, preferably promotes the decay of transcripts with low ribosome occupancies to release ribosomes for further allocation. (**C**) Ribosomes released from the deadenylated mRNAs are reallocated to heavy polysomes to enhance translation efficiency. An example is the enhanced translation produces more MDM2 molecules to suppress p53 activity and avoid cells to be arrested in the G1/G0 phase.

Inconsistency between the changes in mRNA and protein levels is frequently observed in cellular studies of gene expression. We also found that PARN overexpression resulted in opposing effects on the *MDM2* mRNA and protein levels. The poly(A) tail length distribution of the remained *MDM2* mRNAs in the PARN-overexpression group was indistinguishable from the control group. The decreased transcript abundance and increased decay rate implied that the deadenylated *MDM2* transcripts were quickly removed by mRNA degradation machinery. Meanwhile, the remained *MDM2* transcripts were concentrated in heavy polysomes with high ribosome occupancies (Figure 6). These observations led to a proposal that PARN selectively promoted the degradation of the *MDM2* transcripts with relatively low translation efficiency. Ribosomes were released from these deadenylated transcripts and then reallocated to transcripts with high ribosome occupancies. Figure 7 shows a proposed model. By this means, the cells could remove the redundant transcripts to optimize the costs for efficient protein production. This ribosome reallocation mechanism could also explain why the protein levels either increased or unchanged, even though the levels of the five tested cytosolic mRNAs were significantly decreased by overexpressed PARN.

Although PARN predominantly locates in the nucleus, it is a nuclear-cytoplasmic shuttling protein [79]. Herein, we identified that the cytoplasmic PARN can shuttle between the cytosol and ER. Under DNA damaging conditions induced by DOX, the anterior nucleus-localized PARN was quickly translocated to cytoplasm, while the DNA damage-mimic reagent hydroxyl urea did not induce such a translocation [8]. This suggested that the action of PARN during DDR might involve step-wise processes. The step-wise process can also be observed for the action of PARN in the cytoplasm. The ER-anchored PARN had an apex value after 4 h DOX treatment. A possible method is posttranslational modification to achieve such an exquisitely spatiotemporal regulation of PARN activity. The phosphorylation of PARN has been observed in cells with serum starvation [80], DNA damage [66], and pathological conditions, such as acute leukemia [12]. Moreover, Ser557 phosphorylation by MK2 prevents *Gadd45α* mRNA degradation [66]. However, it remains unclear for the underlying mechanism and exact function of PARN phosphorylation. We found that the intrinsically disordered CTD was responsible for the membrane binding of PARN, probably via ionic interactions by the abundant positively charged residues in the CTD. Ser557 is located at the CTD and phosphorylation introduces additional negative charges in the CTD, which probably weaken the ionic interactions between the CTD and ER membrane. The translocation of PARN from the ER to cytosol was strongly dependent on the level of phosphorylated MK2 (Figure 3). Kinase-triggered translocation endows PARN with the potency to respond to diverse signaling pathways. It is worthy noting that the complicated behaviors of the ER-bound PARN imply that the phosphorylation of Ser557 by MK2 is probably only one of the regulators. Further research is needed for identifying the additional regulators modulating the membrane-binding ability of PARN.

Previously, PARN has been shown to participate into DNA damage response (DDR) via multiple pathways, including switching off PARN activity by MAPKAP kinase 2 (MK2)-induced Ser557 phosphorylation to stabilize the *Gadd45α* mRNA in the p38MAPK/MK2 pathway [66], degrading a subset of mRNAs by recruiting to the CstF/BARD1 complex in the nucleus [81], modulating protein-protein interaction network by phosphorylation at S557 [8], and regulating *p53* [74] or *p21* mRNA stability [16]. Herein, we also found that PARN is involved in the p38MAPK/MK2 signaling pathway and identified an additional target of PARN during DDR. ER-anchored PARN decreased *MDM2* mRNA stability, but enhanced its translation efficiency. The enhanced MDM2 protein level sequestered more p53 molecules and inhibited the p53 transcriptional activities, which facilitated the HeLa cells to bypass the checkpoints and thereby promoted cell cycle progression (Figure 6). These findings provide a mechanistic understanding of the delicate control of cell cycle progression by PARN-mediated translation regulation and expand the complicated actions of PARN in DDR and carcinogenesis.

## Supplementary Materials

The following are available online at https://www.mdpi.com/2073-4409/9/1/162/s1, Figure S1: Western blot analysis of PARN subcellular localization in the HeLa cells, HEK-2933T cells and mouse tissues, Figure S2: Sucellular localization of endogenous PARN in the HeLa cells analyzed by confocal microscopy, Figure S3: Membrane insertion ability of αB-crystallin and γD-crystallin, which were taken as the examples of the ER peripheral proteins and exclusively cytosolic proteins., Figure S4: Representative Western blot analysis of PARN translocation in HEK-293T cells induced by DOX treatment and the impact of S557 mutations on the ER localization of PARN, Figure S5: Poly(A) length distribution determined by the Agilent 2100 bioanalyzer through capillary electrophoresis using the Agilent RNA 6000 Pico Kit., Figure S6: Transcriptome analysis and decay kinetics of candidate mRNAs Figure S7: Effect of PARN overexpression on cell cycle progression, poly(A) tail length distribution of *MDM2* and protein levels of cytosolic proteins. Figure S8: Effect of PARN overexpression on the levels of *MDM2* mRNA, PARN, RPS3 and RPL7A in each fraction of the polysome profile, Figure S9: Uncropped western blot images and repletion of Figure 4A.
