# Supplementary material for: Translation Efficiency and Degradation of ER-Associated mRNAs Modulated by ER-Anchored poly(A)-Specific Ribonuclease (PARN)
Source: Cells. 2020 Jan 9;9(1):162. doi: 10.3390/cells9010162 (PMC7017053; doi:10.3390/cells9010162)
Supplement: Supplementary file 1 [file cells-09-00162-s001.pdf]

Supplemental Materials

Translation efficiency and degradation of ER-associated mRNAs modulated by ER-anchored poly(A)-specific ribonuclease (PARN)

Tian-Li Duan, Han Jiao, Guang-Jun He, and Yong-Bin Yan*

State Key Laboratory of Membrane Biology, School of Life Sciences, Tsinghua University, Beijing 100084, China

* **To whom all correspondence should be addressed:** Dr. Yong-Bin Yan, School of Life Sciences, Tsinghua University, Beijing 100084, China; Phone: +86-10-6278-3477; Fax: +86-10-6277-2245; E-mail: ybyan@tsinghua.edu.cn.

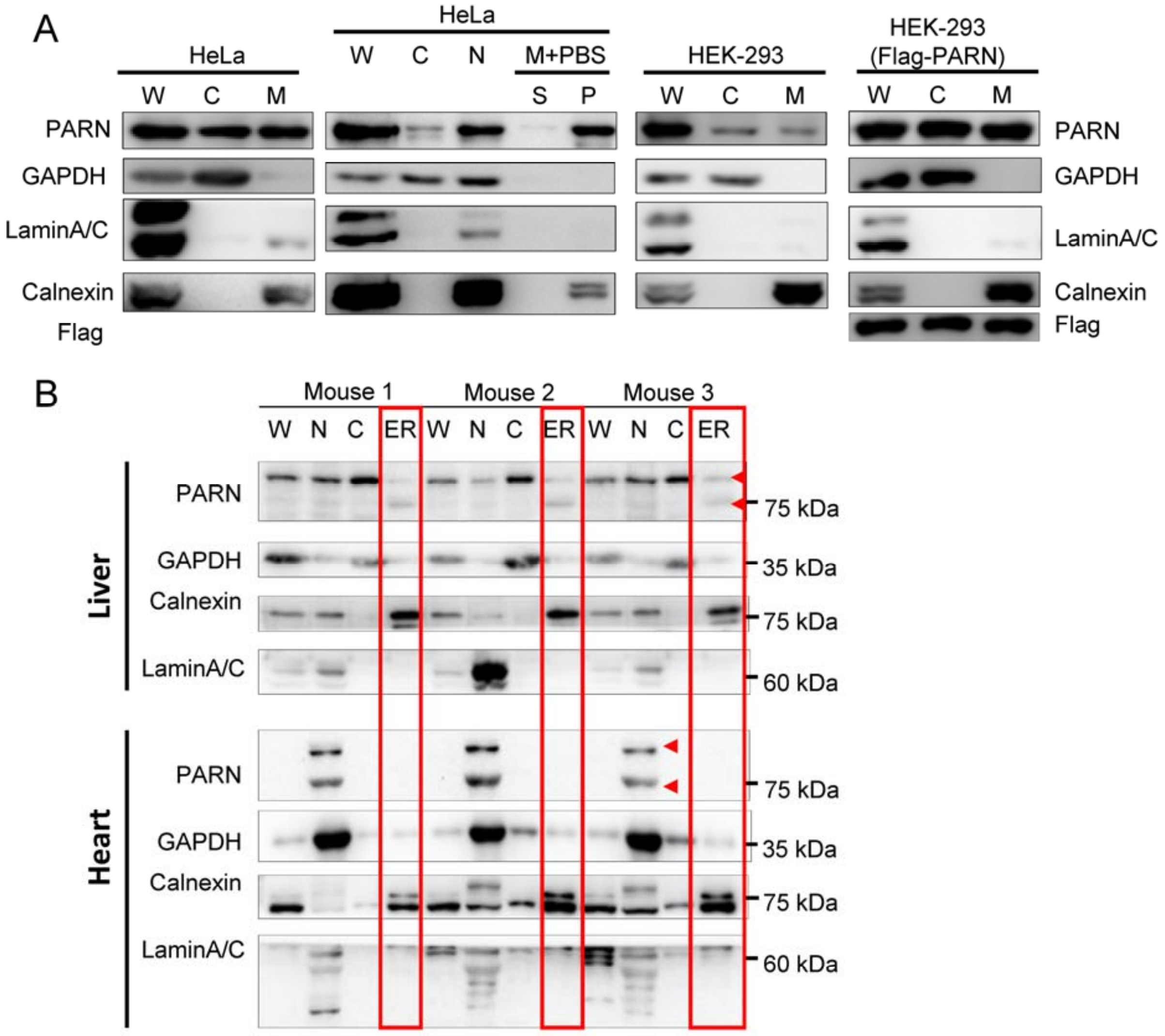

Figure S1. Related to Figure 1. Representative Western blot analysis of PARN subcellular localization in the HeLa cells, HEK-293T cells and mouse tissues. (A) A comparison of the cellular distribution of PARN in the HeLa and HEK-293T cells. The right panel shows the cellular distribution of exogenously overexpressed Flag-PARN in the HEK-293T cells. (B) Subcellular localization of PARN in mouse liver and heart fractionated by differential centrifugation. Quantitative analysis of the data is shown in Figure 1C. A total protein concentration of 30 μg was loaded for the Western blot analysis. The red arrows indicate the presence of two forms of PARN identified by the antibody, while the red rectangles highlight the ER fraction. GAPDH, LaminA/C and calnexin were used as internal markers of cytosol, nucleus and ER, respectively. W, whole lysate; C, cytosol; N, nucleus; M, ER microsomes; S, supernatant; P, precipitation. The presented western blot data were representative ones of three independent experiments.

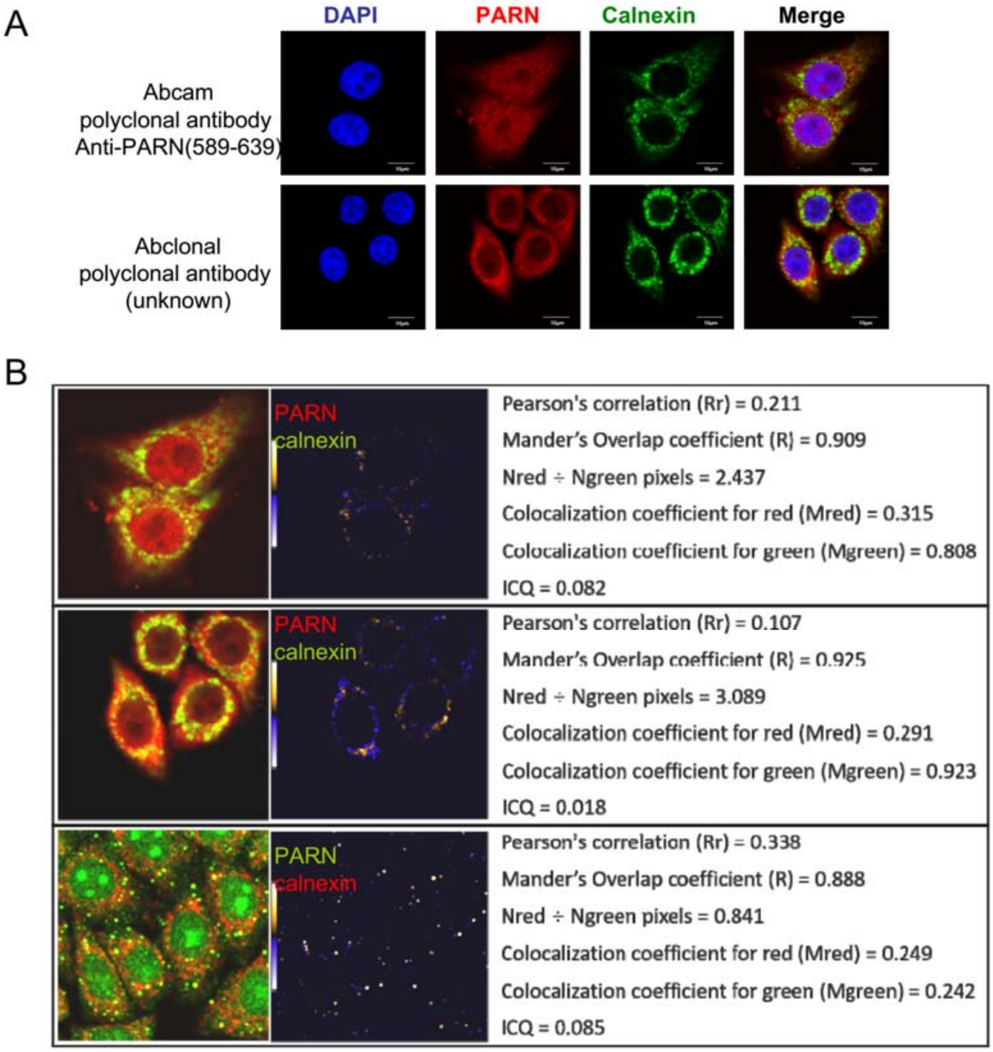

Figure S2. Related to Figure 1. Sucellular localization of endogenous PARN in the HeLa cells analyzed by confocal microscopy. (A) Representative confocal images of endogenous PARN recognized by two commercially available polyclonal antibodies. Dylight 488 and Dylight 594 labeled secondary antibodies were used to hybridize the first antibodies recognizing PARN and calnexin. The nuclei were counterstained with DAPI. The scale bar represents 10 μm. Representative confocal images of endogenous PARN recognized by the monoclonal antibody is shown in Figure 1F. (B) Colocalization analysis of PARN and calnexin for PARN recognized by the three types of antibodies by Image J.

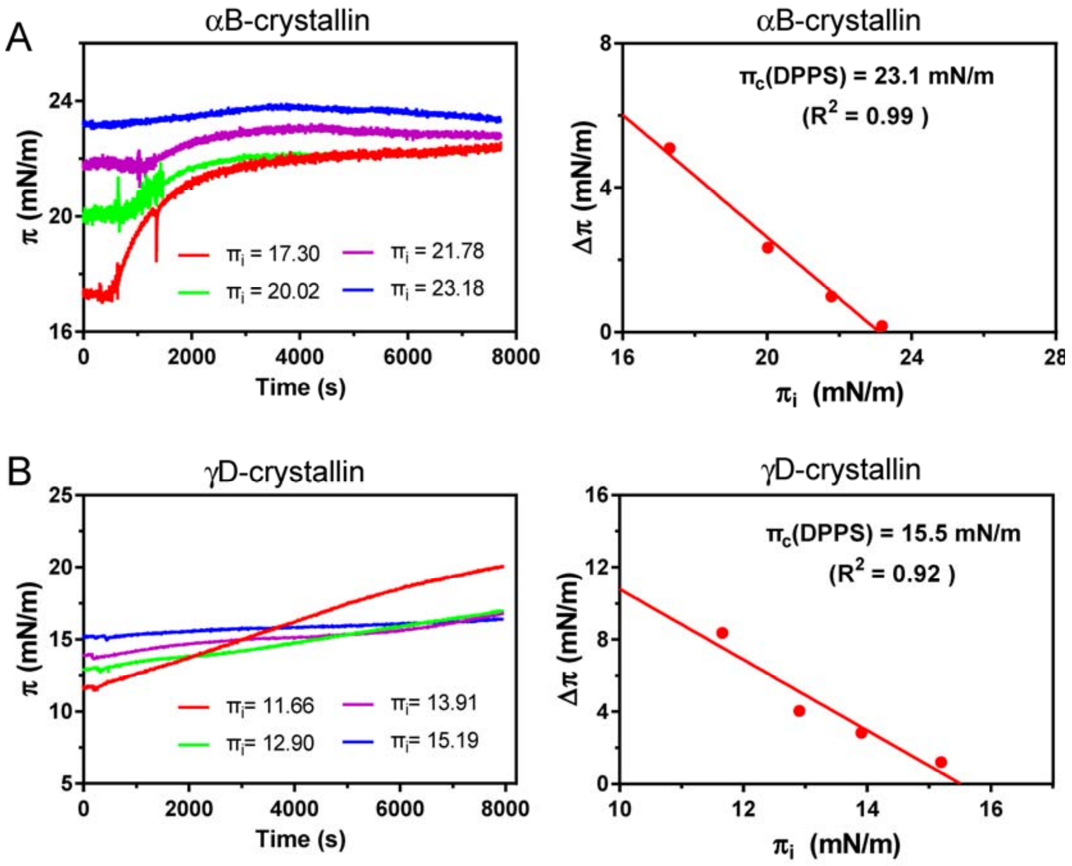

Figure S3. Related to Figure 2. Membrane insertion ability of αB-crystallin (A) and γD-crystallin (B), which were taken as the examples of the ER peripheral proteins and exclusively cytosolic proteins. The experimental conditions were the same as those for PARN shown in Figure 2. Left panel, the time-course changes of surface pressure determined under different initial surface pressure. Right panel, $\Delta\pi$-π_i plots to determine the critical surface pressure π_c.

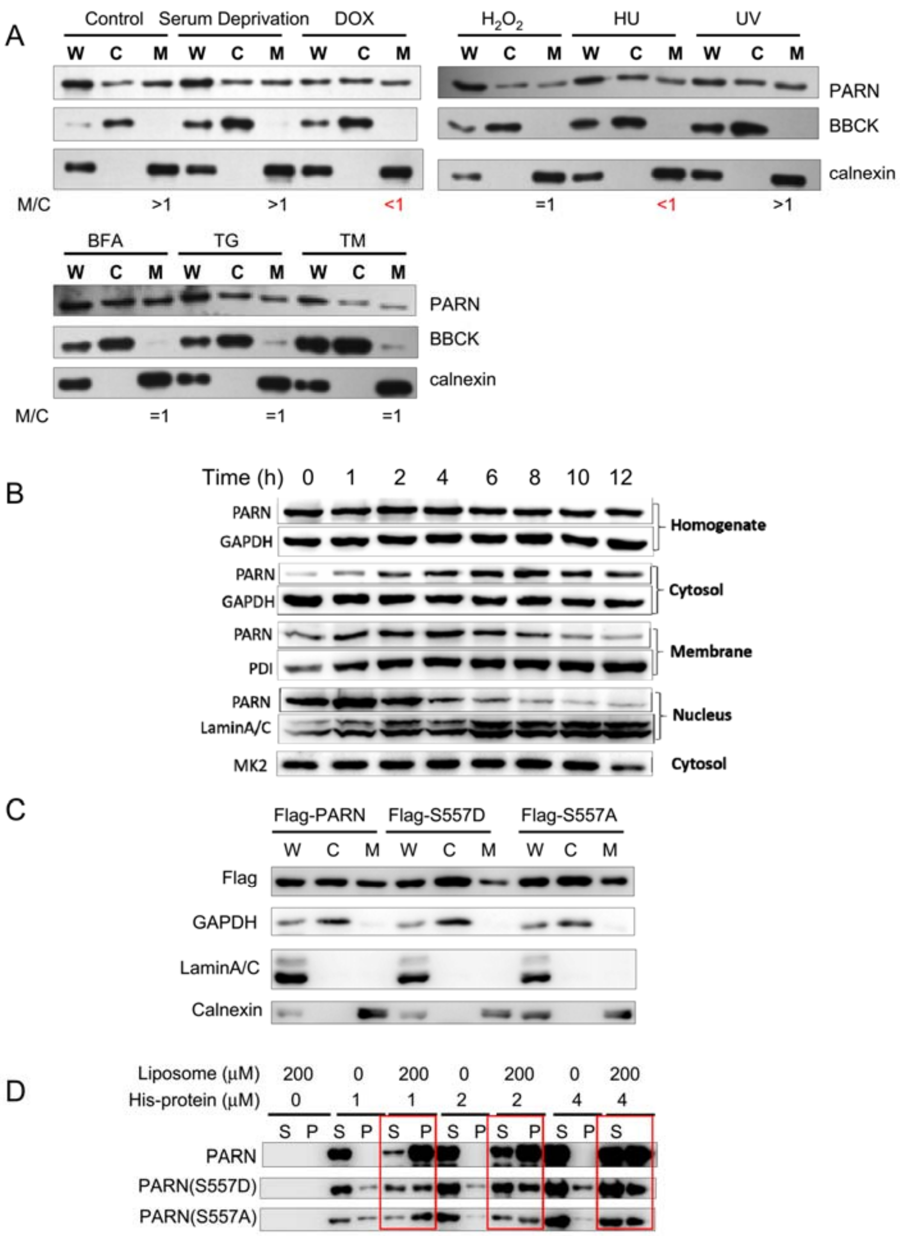

Figure S4. Related to Figure 3. Representative western blot analysis of PARN translocation in the HEK-293T cells induced by DOX treatment and the impact of Ser557 mutations on the ER localization of PARN. (A) A screen of potential factors affecting the membrane association of PARN in the HEK-293T cells. DOX and HU treatments resulted in less amount of membrane-bound PARN, while the other treatments had no significant effects. C/M represents the ratio of gray values between the cytosol band and the ER microsome band. (B) Time-course study of PARN subcellular distribution after treatment of the HEK-293T cells by 10 μM doxorubicin. GAPDH, PDI and lamin A/C were the marker proteins for cytosol, ER and nucleus, respectively. (C) Western blot analysis of overexpressed wild type PARN and its mutants in whole lysates (W), cytosol (C) and ER microsomes (M). (D) Effect of S557D and S557A mutations on the liposome binding ability of purified His-tagged PARN. The presented western blot data were representative ones of three independent experiments. Red rectangles are used to distinguish different groups. S, supernatant; P, precipitation.

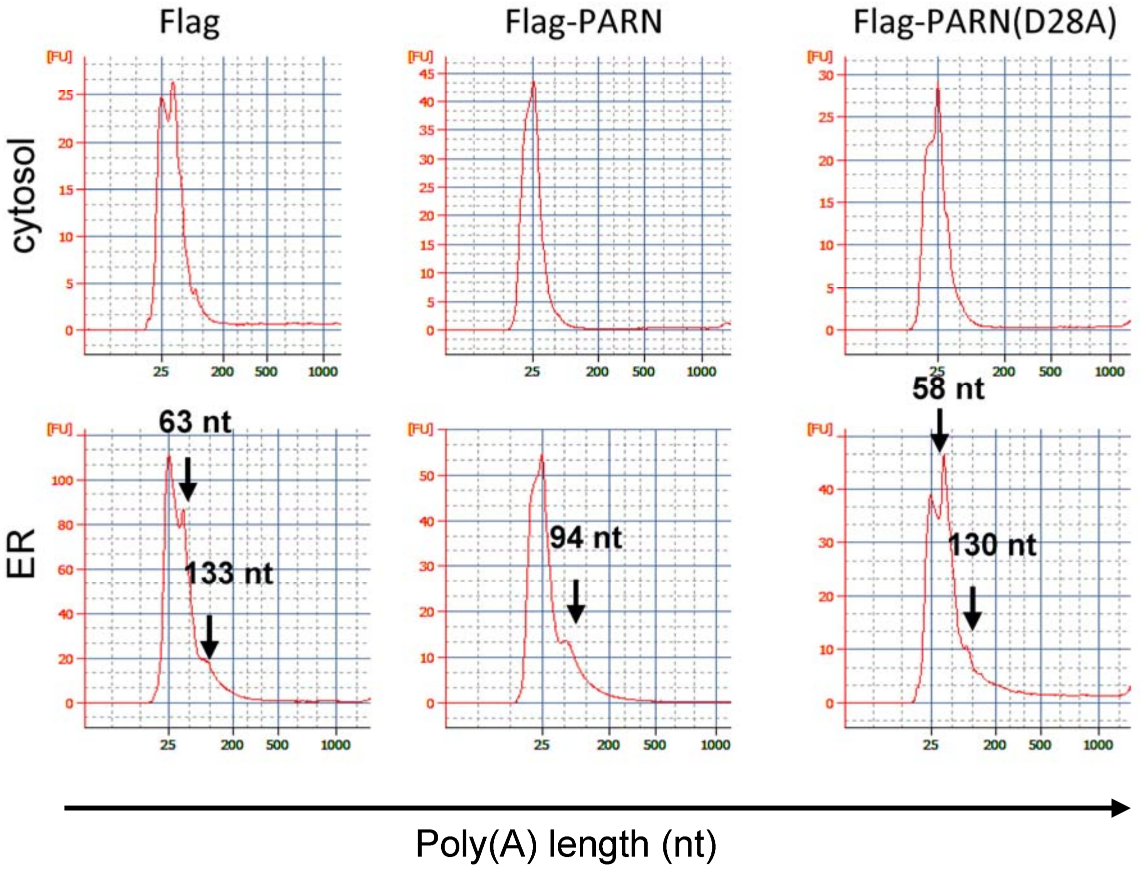

Figure S5. Related to Figure 4. Poly(A) length distribution determined by the Agilent 2100 bioanalyzer through capillary electrophoresis using the Agilent RNA 6000 Pico Kit. The arrows indicate the peak positions and the corresponding length are labeled on top of the arrows.

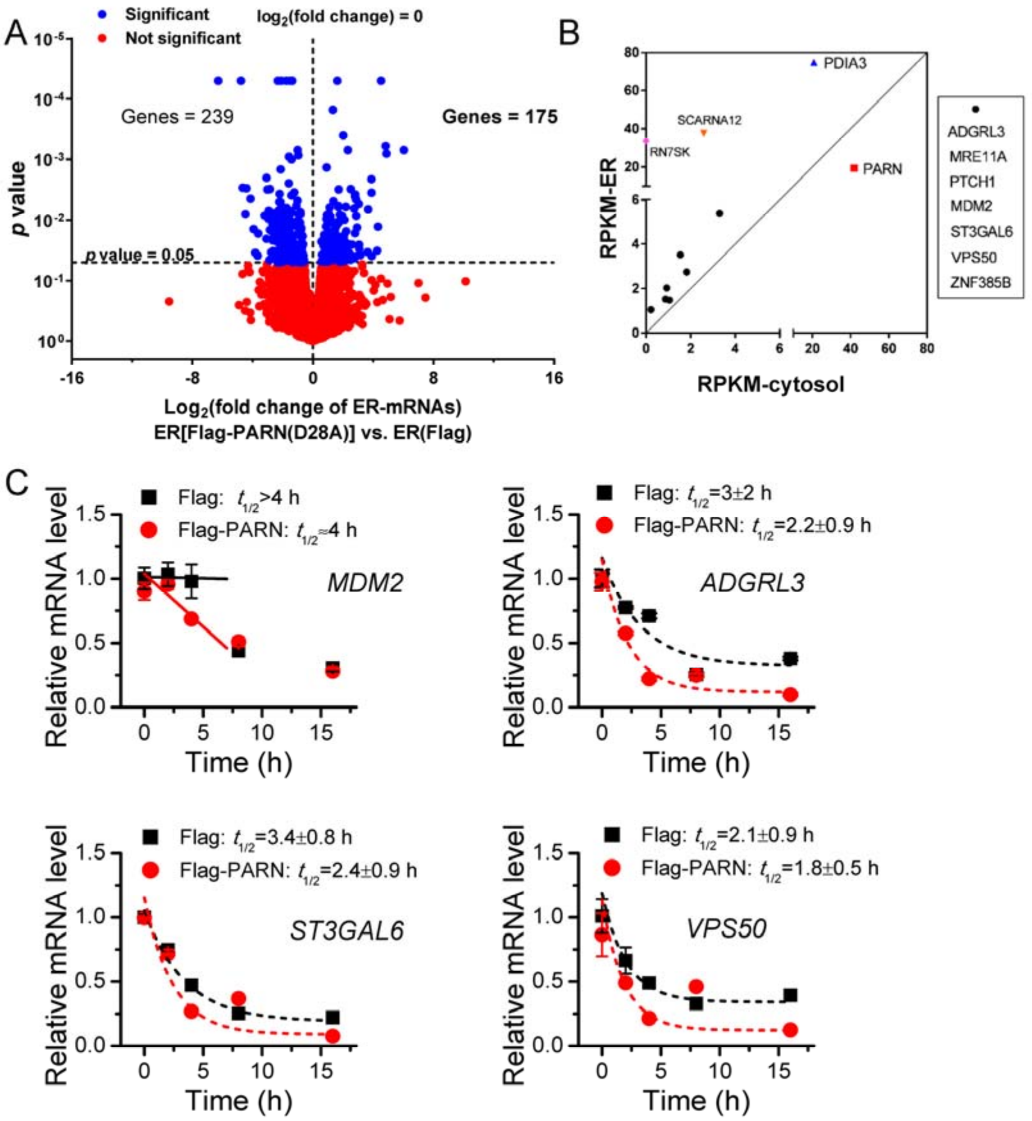

Figure S6. Related to Figure 5. Transcriptome analysis and decay kinetics of candidate mRNAs. (A) The volcano plot of fold change and *p* value for transcripts in the ER fraction of the HEK-293T cells overexpressing wild type PARN vs the inactive mutant PARN(D28A). (B) A comparison of the RPKM values of candidate RNAs in the ER and cytosolic fractions. (C) Effect of PARN overexpression on the decay kinetics of candidate mRNAs (n=3). The half lives of *ADGRL3*, *ST3GAL6* and *VPS50* were obtained by fitting the raw data (symbols) by the single exponential decay kinetics (dashed lines). The decay of *MDM2* did not follow an exponential decay kinetics, which might be caused by the toxic effects of ActD after 5 h treatment. Therefore, the half lives of *MDM2* were estimated from the linear fitting of the data obtained within 5 h.

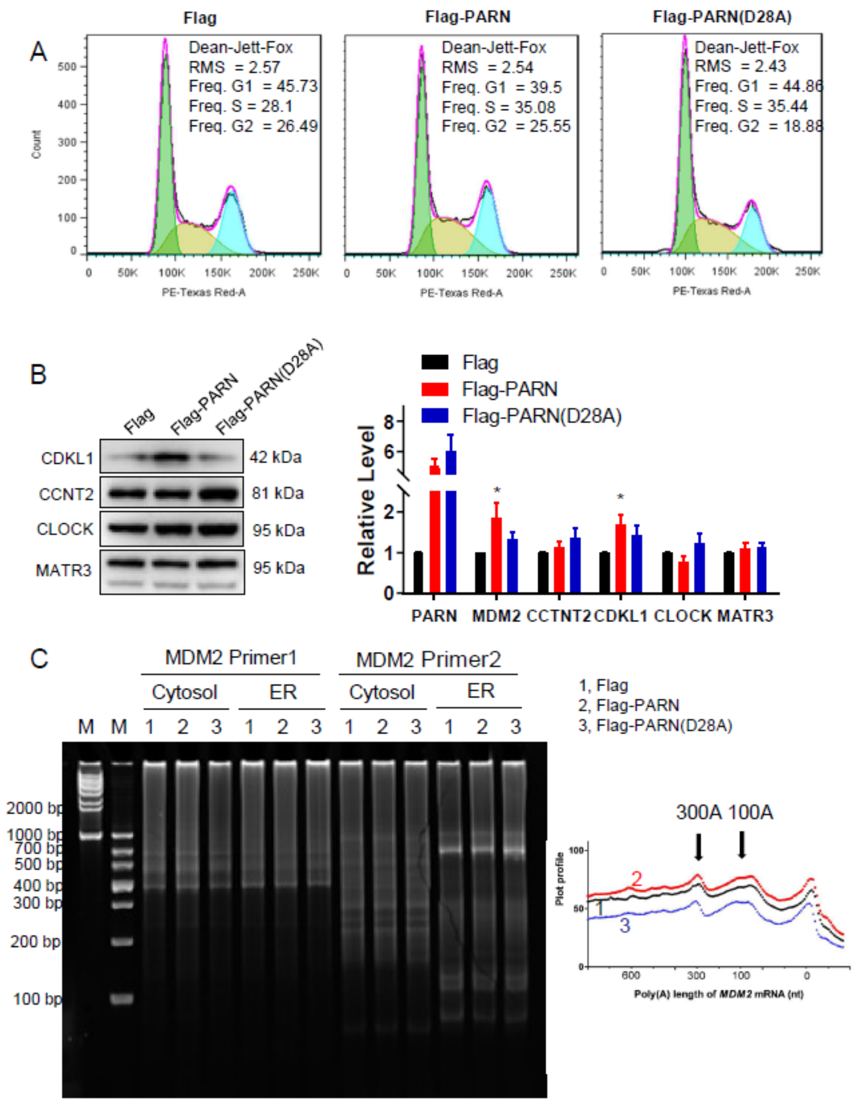

Figure S7. Related to Figure 6. Effect of PARN overexpression on cell cycle progression, poly(A) tail length distribution of *MDM2* and protein levels of cytosolic proteins. (A) Representative cell cycle analysis of the HEK-293T cells overexpressing PARN and its inactive mutant PARN(D28A) by flow cytometer under normal conditions. (B) Effect of PARN overexpression on the protein levels of selected cytosolic proteins translated predominantly in the cytosol. Quantitative analysis is shown in the right panel (n=3). (C) Ligase mediated poly (A) tail test (LM-PAT) of *MDM2* transcripts in the cytosolic and ER fractions extracted from the HEK-293T cells overexpressing PARN or its inactive mutant PARN(D28A). Two primers were designed and named as MDM2 Primer 1 and 2, while Primer 1 showed higher specificity and used for further analysis. The minimal expected amplicons of Primer 1 and 2 are 409 bp and 326 bp, respectively. Samples were resolved on 8% PAGE and stained with GelSafe. 1-3 represents the Flag control, Flag-PARN and Flag-PARN(D28A) groups. Quantification of the bands detected by Primer 1 is shown as the right panel. Two distinct populations centered at around 100A and 300A could be identified.

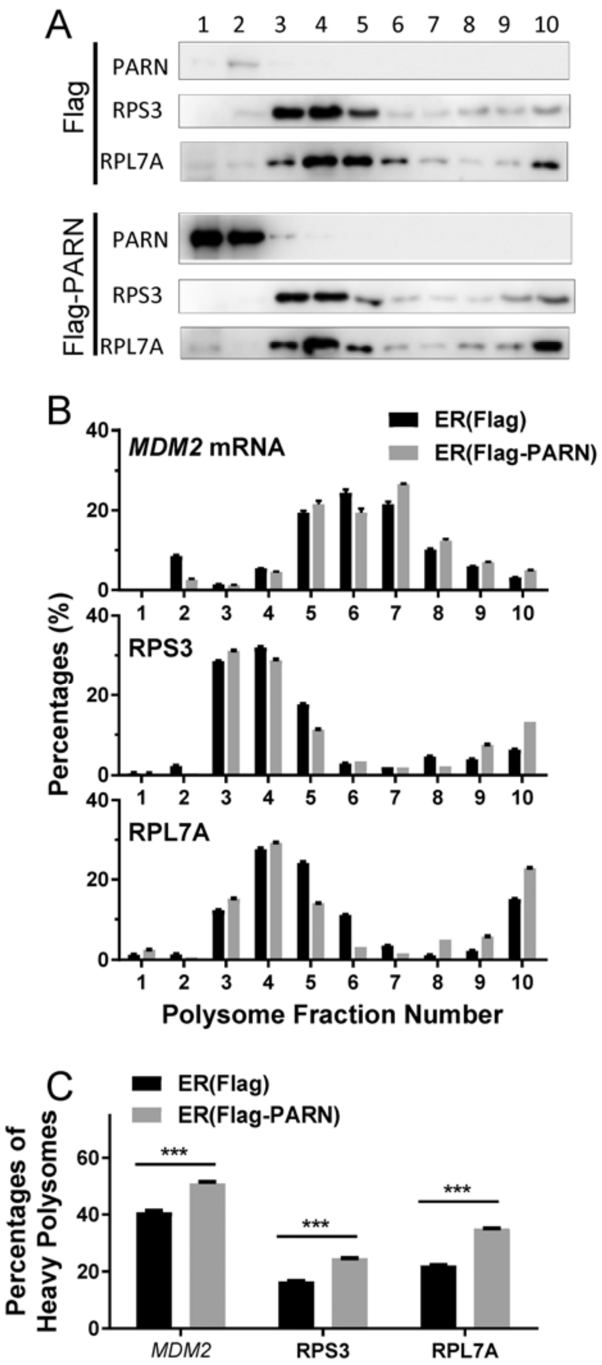

Figure S8. Related to Figure 6. Effect of PARN overexpression on the levels of *MDM2* mRNA, PARN, RPS3 and RPL7A in each fraction of the polysome profile. (A) Representative western blot analysis (n=3). (B) Quantitative analysis of *MDM2* mRNA level, RPS3 and RPL7A protein levels in each fraction of the ER polysome profiles (n=3). *MDM2* mRNA levels were determined by the copy number and a standard curve obtained using *MDM2* fragment. (C) A comparison of the *MDM2* mRNA level, the RPS3 and RPL7A protein levels in heavy polysomes (n=3). *** $p<0.001$.

A: uncropped images of Figure 1A

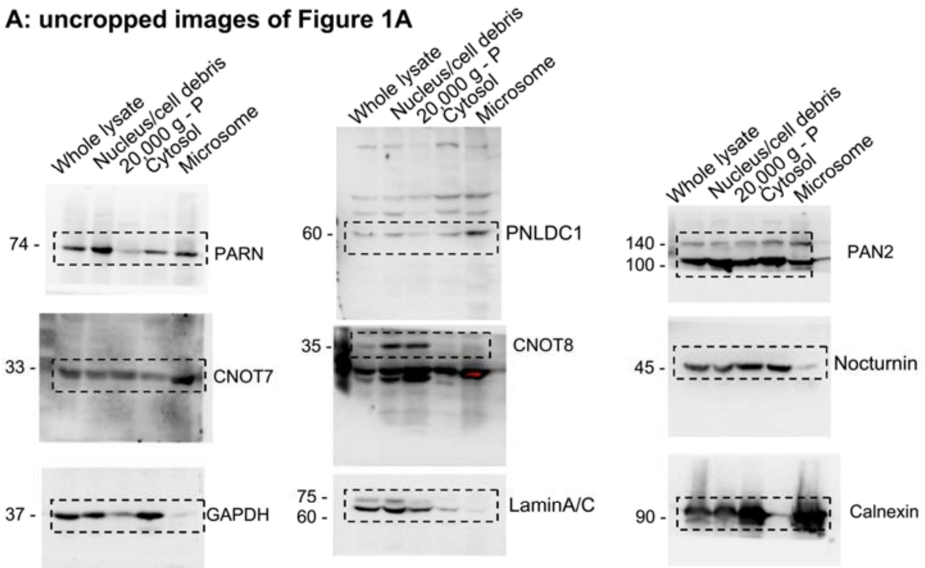

B: uncropped images of Figure 1C

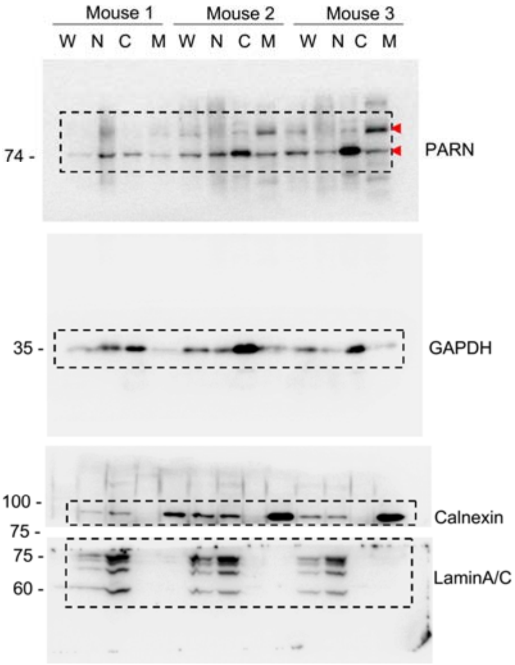

C: uncropped images of Figure 1D

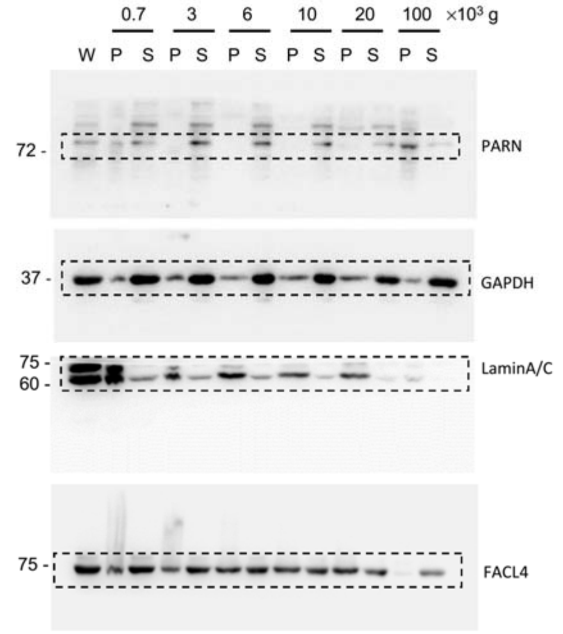

D: uncropped images of Figure 1E

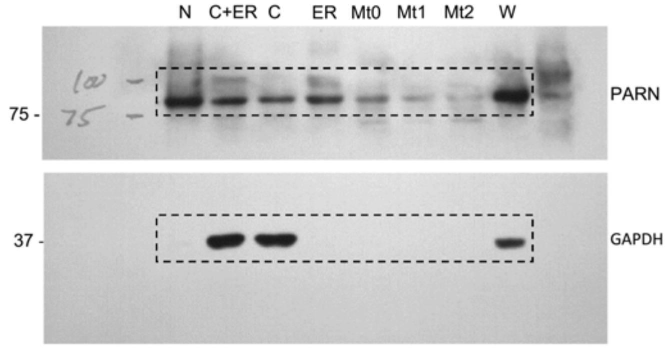

E: uncropped images of Figure 2D

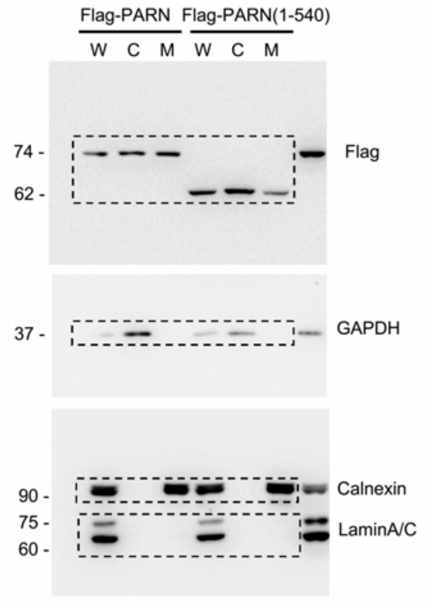

F: uncropped images of Figure 2H

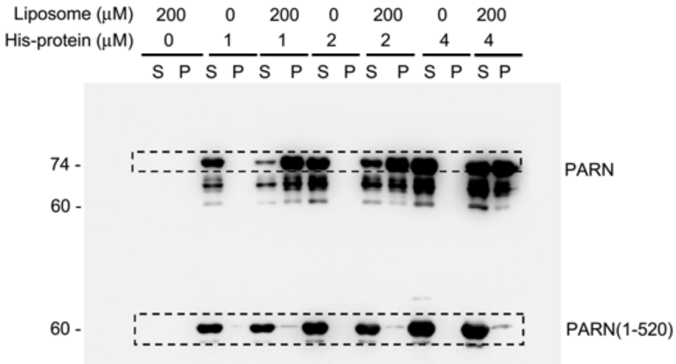

G: uncropped images of Figure 3A

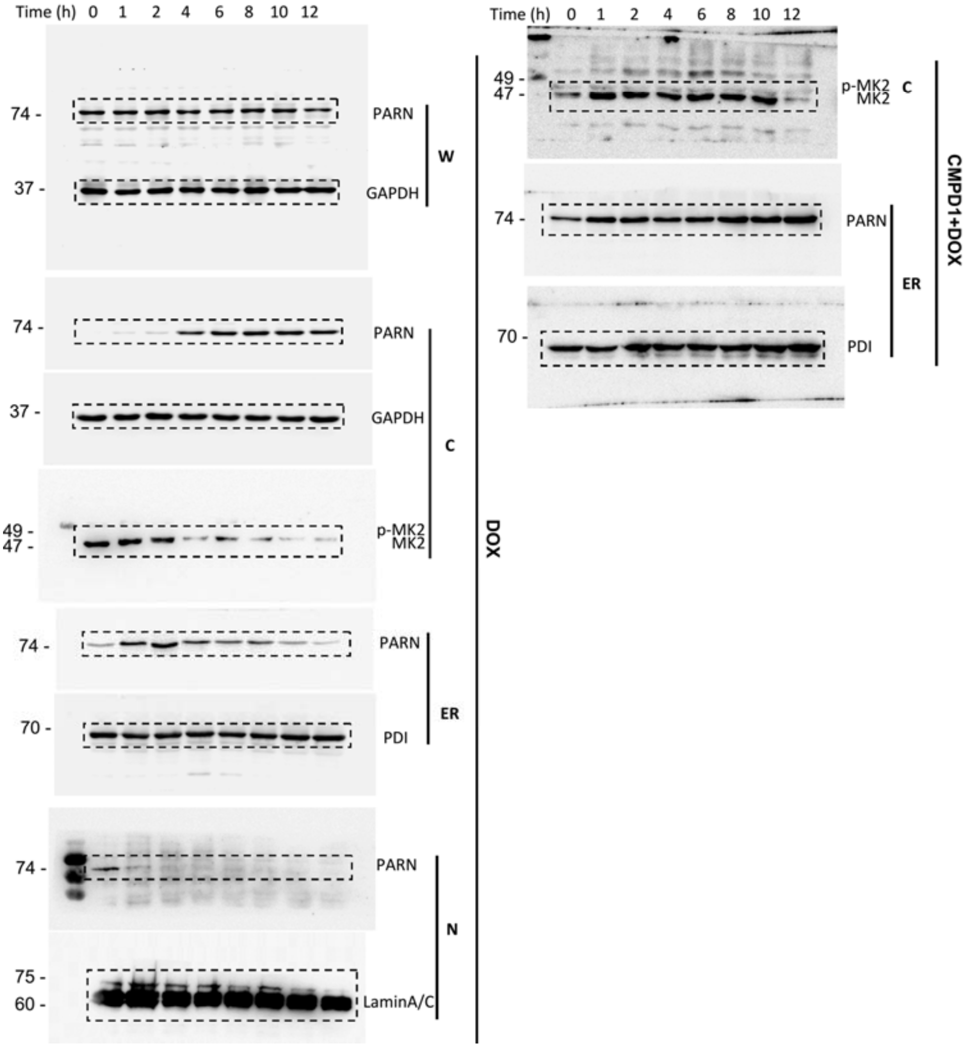

H: uncropped images of Figure 6C

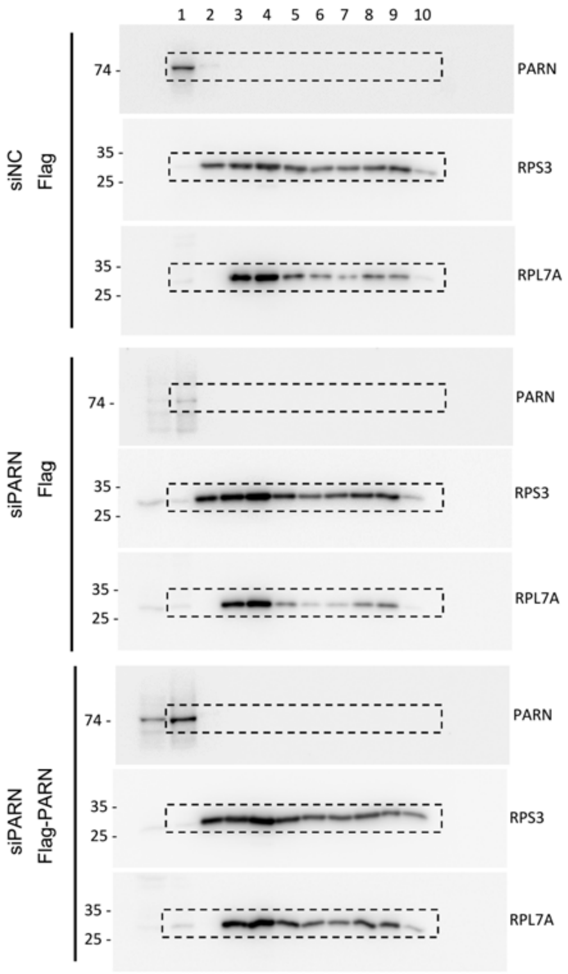

I: Repetition of Figure 4A (on the same gel and the biotinylated RNA band is the same band)

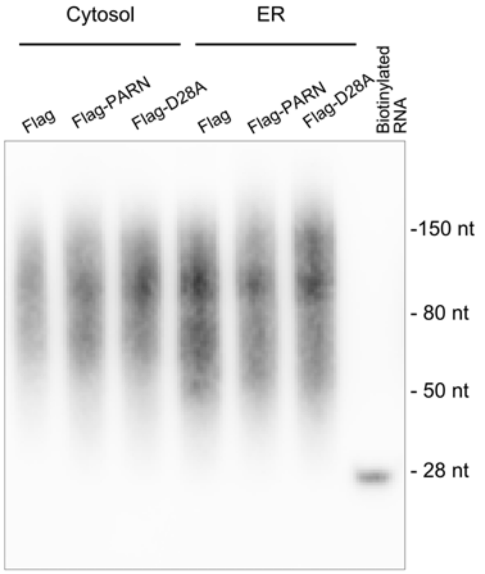

Figure S9. Uncropped western blot images and repletion of Figure 4A. Dotted rectangles indicate the regions used in the corresponding figures.
